# Dual functions of NPC1L1 for cholesterol transport: Cholesterol capture and regulation of inner-leaflet cholesterol accessibility

**DOI:** 10.1016/j.isci.2026.117625

**Published:** 2026-09-24

**Authors:** Yoshihide Yamanashi, Maiko Kitani, Megumi Kumamaru, Tomoki Fukaya, Rina Sugita, Tappei Takada

**Affiliations:** 1 Department of Pharmacy, The University of Tokyo Hospital, 7-3-1 Hongo, Bunkyo-ku, Tokyo 113-8655, Japan; 2 Department of Biopharmaceutics, Graduate School of Pharmaceutical Sciences, Nagoya City University, 3-1 Tanabe-dori, Mizuho-ku, Nagoya, Aichi 467-8603, Japan

**Keywords:** accessible cholesterol, cholesterol uptake, cholesterol probe, transporter, SSD

## Abstract

Niemann-Pick C1-like 1 (NPC1L1) is essential for intestinal cholesterol absorption, but the detailed mechanisms underlying its non-vesicular cholesterol transport remain unclear. Using fluorescent cholesterol probes, we found that NPC1L1 decreased accessible cholesterol in the outer leaflet while increasing it in the inner leaflet in an ezetimibe-sensitive manner. This activity was independent of outer-leaflet sphingomyelin levels and was unaffected by a dysfunctional mutation in the N-terminal domain (NTD), which mediates extracellular cholesterol binding and insertion into the outer leaflet. In contrast, mutations in the sterol-sensing domain (SSD) abolished NPC1L1-dependent redistribution of accessible cholesterol between plasma membrane leaflets. These findings support a two-step model in which the NTD captures and inserts cholesterol into the outer leaflet, whereas the SSD promotes its redistribution toward the inner leaflet. Our study identifies a previously unrecognized step in NPC1L1-mediated non-vesicular cholesterol transport and provides mechanistic insight into SSD function.

## Highlights

• NPC1L1 mediates non-vesicular cholesterol uptake through its NTD and SSD
• NPC1L1 inserts micellar cholesterol into the outer leaflet via its NTD
• NPC1L1 facilitates cholesterol distribution to the inner leaflet via its SSD
• Ezetimibe inhibits both NTD- and SSD-mediated functions of NPC1L1

## Graphical abstract

**Figure:**
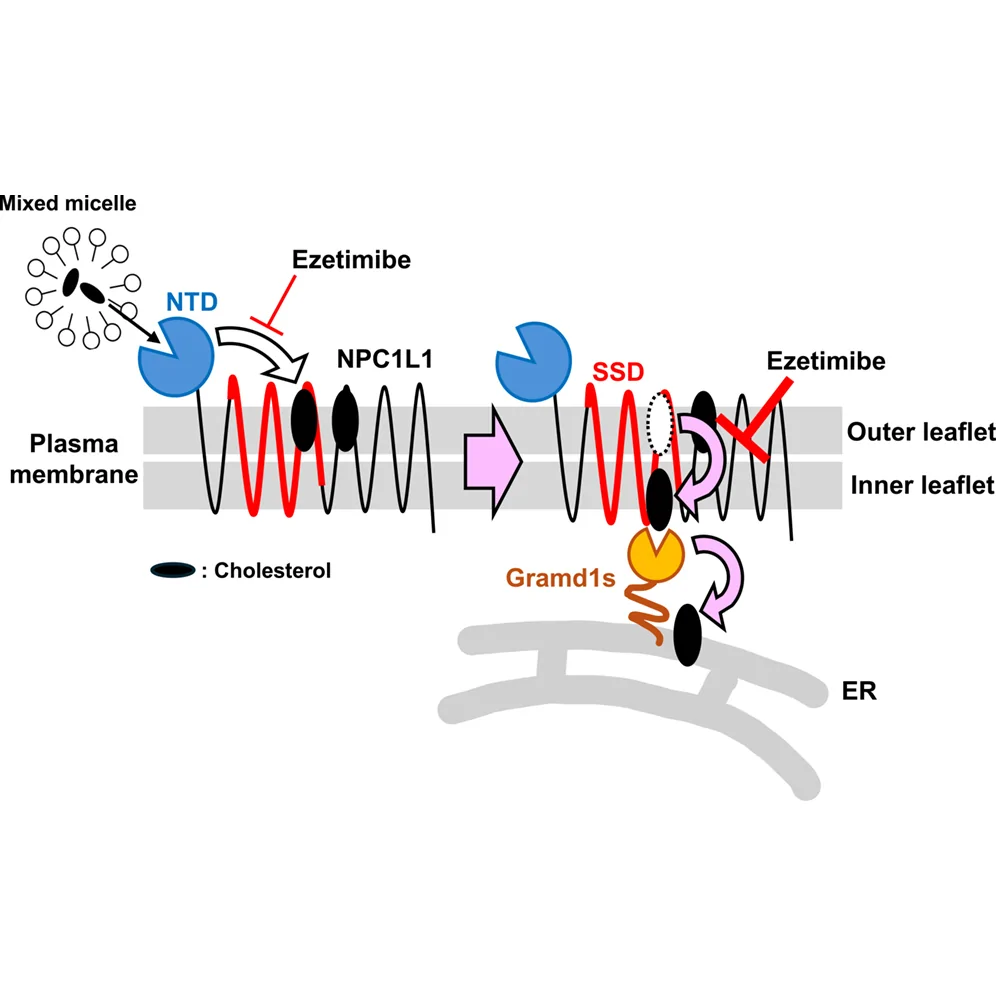

## Teaser

Biological sciences; Biochemistry; Membranes; Cell biology

## Introduction

Cholesterol is an essential molecule in bodily processes; however, increased blood cholesterol levels are a risk factor for cardiovascular events such as atherosclerosis and myocardial infarction. For maintaining cholesterol homeostasis, dietary cholesterol absorption is important.^1^ In 2004, Altmann et al. reported that cholesterol absorption decreased by 70% in Niemann-Pick C1 (NPC1)-like 1 (NPC1L1)-knockout (KO) mice, indicating that NPC1L1 plays a central role in cholesterol absorption in the intestine.^2^ In addition, NPC1L1 is known to be the target protein of ezetimibe, a cholesterol absorption inhibitor clinically used for the treatment of dyslipidemia.^2^^,^^3^ Thus, from (patho)physiological and pharmacological perspectives, NPC1L1 is considered to be an important protein.

Structurally, NPC1L1 contains a sterol-sensing domain (SSD), a highly conserved transmembrane region shared with other key regulators of cholesterol metabolism, such as NPC1 and 3-hydroxy-3-methylglutaryl coenzyme A (HMG-CoA) reductase. Although NPC1L1 has been extensively studied in the context of vesicular endocytic cholesterol uptake,^4^^,^^5^^,^^6^^,^^7^ a recent study identified an additional role for NPC1L1 in non-vesicular cholesterol uptake.^8^^,^^9^^,^^10^^,^^11^^,^^12^^,^^13^^,^^14^ However, detailed mechanisms by which extracellular cholesterol crosses the plasma membrane (PM) during the non-vesicular process remain unclear, particularly with respect to the role of the SSD.

Recently, it was proposed that cholesterol in the cell membrane could be biochemically categorized into two distinct states based on its interactions with other lipids and proteins.^15^ One is “accessible cholesterol,” which is freely available for interaction with proteins, and the other is “inaccessible cholesterol,” which is sequestered by membrane phospholipids such as sphingomyelin. Among these, accessible cholesterol is important for cholesterol transport between organelles, including the PM, via intracellular lipid transfer proteins such as oxysterol-binding protein-related proteins and GRAMD family proteins.^16^^,^^17^

Recent advances on cholesterol probes utilizing the domain 4 (D4) of perfringolysin O (PFO, cholesterol-binding theta toxin) and its mutant (D434S), known as D4H, which has a higher affinity for cholesterol, have enabled us to visualize and quantify accessible cholesterol in the outer and inner leaflets of the cell membrane individually.^18^ Although cholesterol has been shown to be able to flip-flop quickly and spontaneously across the lipid bilayer,^19^ recent studies suggest that an asymmetric distribution of cholesterol can nonetheless be established within the PM by sterol efflux transporters and phospholipid contents in the PM. Indeed, although some studies have reported differing conclusions,^20^^,^^21^ recent studies using D4-based probes have demonstrated that cholesterol levels in the PM are generally higher in the outer leaflets than in the inner leaflets of mammalian cells,^22^ a finding further supported by a recent report employing a fluorescent cholesterol analog.^23^

Considering this background, we hypothesized that efficient non-vesicular transport of cholesterol from the PM to intracellular organelles during the process of cholesterol absorption (uptake) requires redistribution of cholesterol between PM leaflets, thereby increasing its accessibility to intracellular proteins (organelles). We therefore investigated whether NPC1L1 influences the distribution of accessible cholesterol between the outer and inner leaflets of the PM. By image analysis of accessible cholesterol using fluorescent protein-tagged D4(H) probes, we found that membrane expression of NPC1L1 reduced accessible cholesterol in the outer leaflet, whereas accessible cholesterol in the inner leaflet increased in an ezetimibe-sensitive manner. Notably, this activity was abolished by mutations in the SSD, which have previously been reported to decrease cholesterol uptake by NPC1L1. These findings identify a previously unrecognized role of NPC1L1 in regulating the distribution of accessible cholesterol between PM leaflets and provide novel mechanistic insights into the function of the SSD in NPC1L1-mediated cholesterol transport.

## Results

### Cholesterol uptake increases by overexpressing NPC1L1 in McA-RH7777 cells

To confirm whether NPC1L1 exhibits cholesterol uptake activity in McA-RH7777 cells, a micellar cholesterol uptake assay was performed. Consistent with previous reports,^8^^,^^11^ NPC1L1-expressing cells exhibited increased cholesterol uptake (Figure S1). Moreover, this increase was inhibited by ezetimibe. These results indicate that NPC1L1 functions as an ezetimibe-sensitive cholesterol uptake transporter in McA-RH7777 cells. In subsequent experiments, we used McA-RH7777 cells to examine whether NPC1L1 altered the accessible cholesterol balance between lipid bilayers of the PM.

### D4 and D4H can work as cholesterol probes in McA-RH7777 cells

To visualize accessible cholesterol in the outer leaflet of the PM, we used purified mCherry-tagged D4 protein (mCherry-D4) and GFP-tagged D4 protein (Figures S2A–S2C). When McA-RH7777 cells were incubated with mCherry-D4 or GFP-D4, the cell membranes were outlined with red or green fluorescence derived from the respective probes (Figures S2D and S2E). Meanwhile, cells depleted of cholesterol by pretreatment with methyl-β-cyclodextrin (MβCD) exhibited negligible mCherry-D4 or GFP-D4 fluorescence (Figures S2D and S2E), confirming that purified mCherry-D4 and GFP-D4 proteins function as cholesterol probes in the outer leaflet of the PM.

To visualize the inner leaflet of accessible cholesterol, we applied mCherry-D4H as reported previously by Maekawa and Farin^24^ (Figure S2F). McA-RH7777 cells were transfected with an mCherry-D4H-expressing vector, and 48 h after transfection, red fluorescence emitting from mCherry-D4H was observed in the PM, in addition to some intracellular compartments (Figure S2G). When membrane cholesterol was depleted by pretreatment with MβCD, red fluorescence was barely observed at the PM (Figure S2G), confirming that mCherry-D4H functions as a probe for accessible cholesterol in the inner leaflet of the PM.

### NPC1L1 decreases accessible cholesterol in the outer leaflet of the PM

To determine whether NPC1L1 regulates accessible cholesterol distribution between lipid bilayers of the PM, we visualized cholesterol in the outer and inner leaflets using mCherry-D4 and mCherry-D4H, respectively. Analyses were performed only in cells in which NPC1L1 was localized in the PM, and subsequent analyses using D4 and D4H probes were conducted accordingly. First, we elucidated the effects of NPC1L1 on accessible cholesterol in the outer leaflet and found that NPC1L1-expressing cells exhibited significantly reduced mCherry-D4-derived red fluorescence intensity compared to control GFP-expressing cells (Figures 1A and 1B). This decrease was abolished when NPC1L1-expressing cells were pretreated with ezetimibe for 24 h in a concentration-dependent manner, with inhibition becoming evident at ∼2 μM and reaching near-maximal levels at ∼10 μM (Figures 1A and 1B; Figure S3A). In contrast, ezetimibe treatment did not alter mCherry-D4 fluorescent in GFP-expressing control cells (Figures 1A and 1B), indicating that the observed effect was NPC1L1-dependent rather than a consequence of nonspecific changes in membrane lipid organization induced by ezetimibe. We confirmed by western blot analyses and cell-surface biotinylation assays that ezetimibe treatment had little effect on either the total protein expression or the PM expression of NPC1L1 under the experimental condition (Figure S4). In addition, ezetimibe was able to reverse this decrease even with only a 1-h short-term treatment (Figures S5A and S5B), further supporting that its effect is unlikely to be indirect or secondary. These results indicate that NPC1L1 reduces accessible cholesterol in the outer leaflet of the PM in an ezetimibe-sensitive manner.

**Figure 1:**
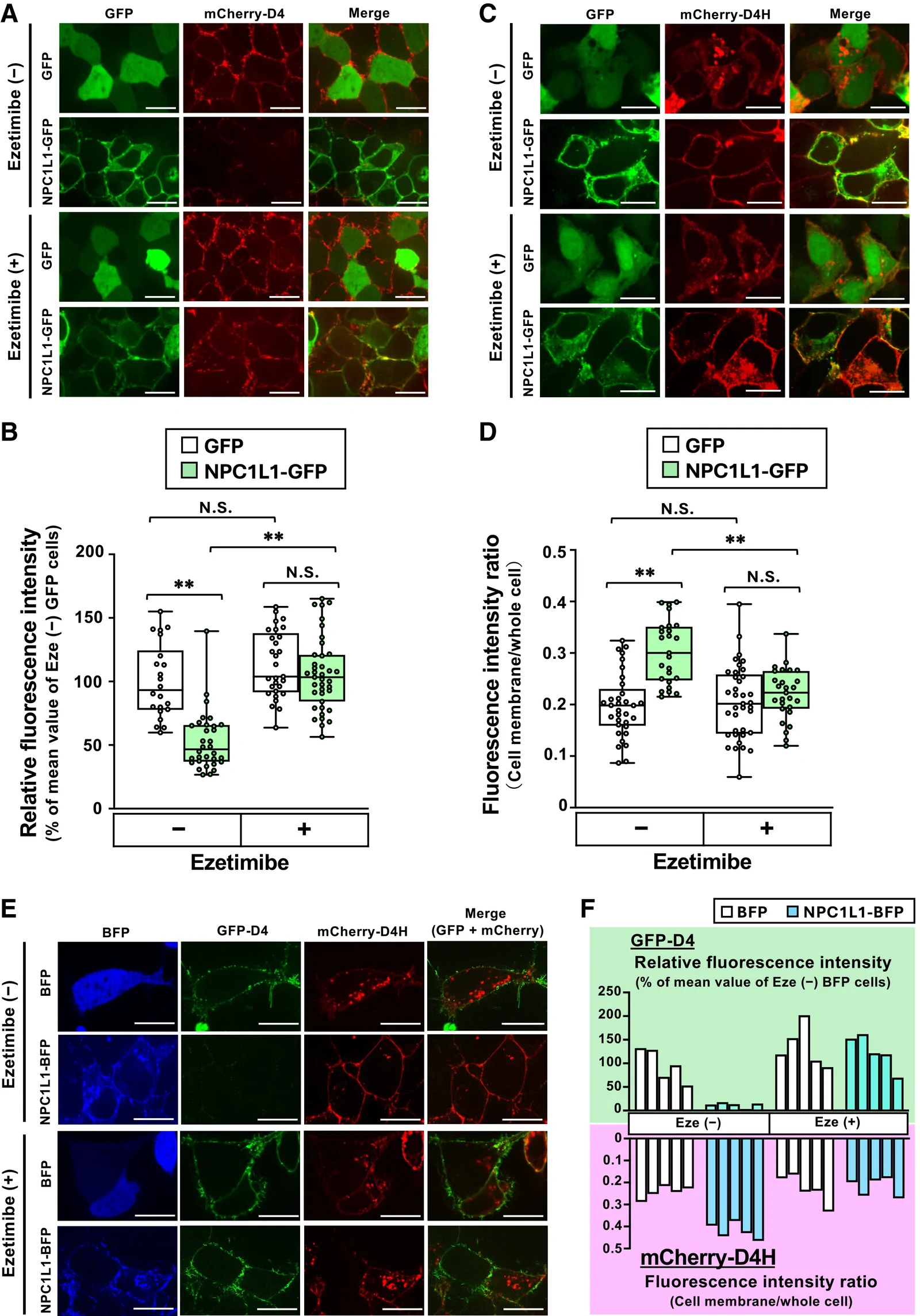
Effect of NPC1L1 on the cholesterol distribution in lipid bilayers of the plasma membrane (A) McA-RH7777 cells were transfected with either GFP-expressing vector (control) or NPC1L1-GFP-expressing vector. Twenty-four hours after transfection, the cells were incubated for an additional 24 h in the absence (−) or presence (+) of 30 μM ezetimibe, followed by labeling with the recombinant mCherry-D4 probe for 15 min. Representative images of GFP fluorescence, mCherry fluorescence, and merged composites are shown. Scale bars represent 20 μm. (B) Quantification of intensity of mCherry fluorescence on the plasma membrane (PM). Relative fluorescence intensity compared with the mean value of ezetimibe-untreated GFP-expressing cells (Eze (−), GFP cells) is shown (*n* = 22 [Eze (−), GFP cells], 32 [Eze (−) and NPC1L1-GFP cells], 29 [Eze (+), GFP cells], and 39 [Eze (+), NPC1L1-GFP cells]). (C) McA-RH7777 cells were co-transfected with mCherry-D4H-expressing vector and either GFP- or NPC1L1-GFP-expressing vector. Twenty-four hours after transfection, the cells were further incubated for 24 h in the absence (−) or presence (+) of 30 μM ezetimibe. Representative images of GFP fluorescence, mCherry fluorescence, and merged composites are shown. Scale bars represent 20 μm. (D) Quantification of mCherry fluorescence intensity at the PM. Ratio of fluorescence intensity calculated as mCherry fluorescence intensity at the PM divided by that of the whole cell is shown (*n* = 33 [Eze (−), GFP cells], 25 [Eze (−), NPC1L1-GFP cells], 37 [Eze (+), GFP cells], and 27 [Eze (+), NPC1L1-GFP cells]). Box and whiskers plots represent median (line in box center), first and third quartiles (lower and upper box border, respectively), and minimum and maximum values (whiskers). Dots represent individual data points. ∗∗*p* < 0.01, significantly different using Sidak’s multiple comparison test. N.S., not significantly different. (E) McA-RH7777 cells were co-transfected with mCherry-D4H-expressing vector and either BFP- or NPC1L1-BFP-expressing vector. Twenty-four hours after transfection, the cells were further incubated for 24 h in the absence (−) or presence (+) of 30 μM ezetimibe, followed by labeling with the recombinant GFP-D4 probe for 15 min. Representative images of BFP fluorescence, GFP fluorescence, mCherry fluorescence, and merged composites of GFP and mCherry fluorescence are shown. Scale bars represent 20 μm. (F) Quantification of intensity of GFP fluorescence on the plasma membrane (PM). Relative fluorescence intensity compared with the mean value of ezetimibe-untreated BFP-expressing cells (Eze (−), BFP cells) is shown (top). Ratio of fluorescence intensity calculated as mCherry fluorescence intensity at the PM divided by that of the whole cell is shown (bottom). Data from five individual cells under each indicated condition are presented separately.

### NPC1L1 increases accessible cholesterol in the inner leaflet of the PM

Next, to elucidate the effects of NPC1L1 on accessible cholesterol in the inner leaflet of the PM, mCherry-D4H-expressing vector was co-transfected with NPC1L1- or GFP-expressing vectors into McA-RH7777 cells. Control GFP-expressing cells exhibited mCherry-D4H-derived red fluorescence in both the PM and intracellular compartments; however, NPC1L1-expressing cells exhibited stronger red fluorescence in the PM than in the intracellular compartments (Figure 1C). Because the fluorescence intensity emitted from mCherry-D4H is affected by transfection efficiency of the mCherry-D4H-expressing vector in each cell, the influence of NPC1L1 was evaluated using the PM fluorescence ratio—a corrected value obtained by dividing fluorescence intensity of the PM by that of whole cell. Although the whole-cell mCherry fluorescence intensity did not differ significantly between control and NPC1L1-expressing cells (Figure S6A), the ratio of PM to whole-cell fluorescence was significantly higher in NPC1L1-expressing cells than in control cells (Figures 1C and 1D). This increase was abolished by ezetimibe treatment. Similar to the results obtained with mCherry-D4, the inhibitory effect of ezetimibe was concentration-dependent and was not observed in GFP-expressing control cells (Figure S3B; Figures 1C and 1D). Furthermore, short-term (1 h) ezetimibe treatment was sufficient to prevent the increase in the PM fluorescence ratio (Figures S5C and S5D). To exclude the possibility that these findings were influenced by differences in cellular mCherry-D4H expression levels, cells were separately analyzed according to high and low intracellular mCherry-D4H fluorescence intensity. NPC1L1 significantly increased the PM fluorescence ratio in both populations, and this effect was similarly abolished by ezetimibe treatment (Figure S7A), indicating that the observed changes were independent of probe expression levels.

To further validate these findings using a different cholesterol probe, we employed cells expressing GFP-GRAM-W, which was developed recently and specifically detects accessible cholesterol in the inner leaflet of the PM.^25^ Consistent with the mCherry-D4H results, NPC1L1-BFP expression significantly increased PM-localized GFP-GRAM-W fluorescence, and this increase was suppressed by ezetimibe treatment (Figure S8). Collectively, these results indicate that NPC1L1 increases accessible cholesterol in the inner leaflet of the PM in an ezetimibe-sensitive manner.

### NPC1L1 regulates accessible cholesterol distribution across PM leaflets

We further performed experiments in which both GFP-D4 and mCherry-D4H probes were applied simultaneously to the same live cells (either BFP-expressing control cells or NPC1L1-BFP-expressing cells) (Figures 1E and 1F; Figure S9). Consistent with the results obtained from the separate staining experiments, NPC1L1-BFP expression markedly decreased GFP-D4 fluorescence intensity and increased the mCherry-D4H fluorescence ratio, indicating a redistribution of cholesterol from the outer to the inner leaflet. These changes were abolished by ezetimibe treatment, further supporting a role for NPC1L1 in regulating accessible cholesterol distribution across PM leaflets in an ezetimibe-sensitive manner.

However, as a potential concern regarding the co-staining approach, we found that overall GFP-D4 fluorescence intensity tended to decrease when mCherry-D4H was expressed (Figure S10), and GFP-D4 fluorescence was barely detectable—particularly in cells co-expressing NPC1L1-BFP and mCherry-D4H (Figures 1E and 1F; Figure S9A). This observation suggests that expression of D4H itself may influence D4-based measurements of cholesterol accessibility in the outer leaflet. To avoid such potential interference, we concluded that it would be more appropriate to analyze D4 and D4H probes separately in all subsequent experiments.

### NPC1L1 has minimal impact on distribution of sphingomyelin in the outer leaflet of the PM

Sphingomyelin is one of the sphingolipids that are abundantly present in the outer leaflet of PM and has been reported to regulate the amount and/or accessibility of cholesterol by interacting (binding) with cholesterol in the PM. Taken together with our previous observations that NPC1L1 has sphingomyelin uptake activity,^26^ it is possible that NPC1L1-mediated regulation of accessible cholesterol distribution across PM leaflets is a secondary effect of altered sphingomyelin levels in the outer leaflet of PM. To test this, we analyzed sphingomyelin distribution (levels) in the PM by using purified mCherry-tagged non-toxic lysenin (mCherry-NT-Lys), which allows visualization of sphingomyelin in the outer leaflet of PM.^27^ We observed no significant differences in the intensity of red fluorescence emitted from mCherry-NT-Lys between NPC1L1-expressing and control GFP-expressing cells (Figures 2A and 2B), indicating that NPC1L1 had little effect on the distribution of sphingomyelin in the outer leaflet of the PM. These results suggest that NPC1L1-mediated changes in the balance of accessible cholesterol in lipid bilayers are not due to reduced levels (or altered distribution) of sphingomyelin in the outer leaflet.

**Figure 2:**
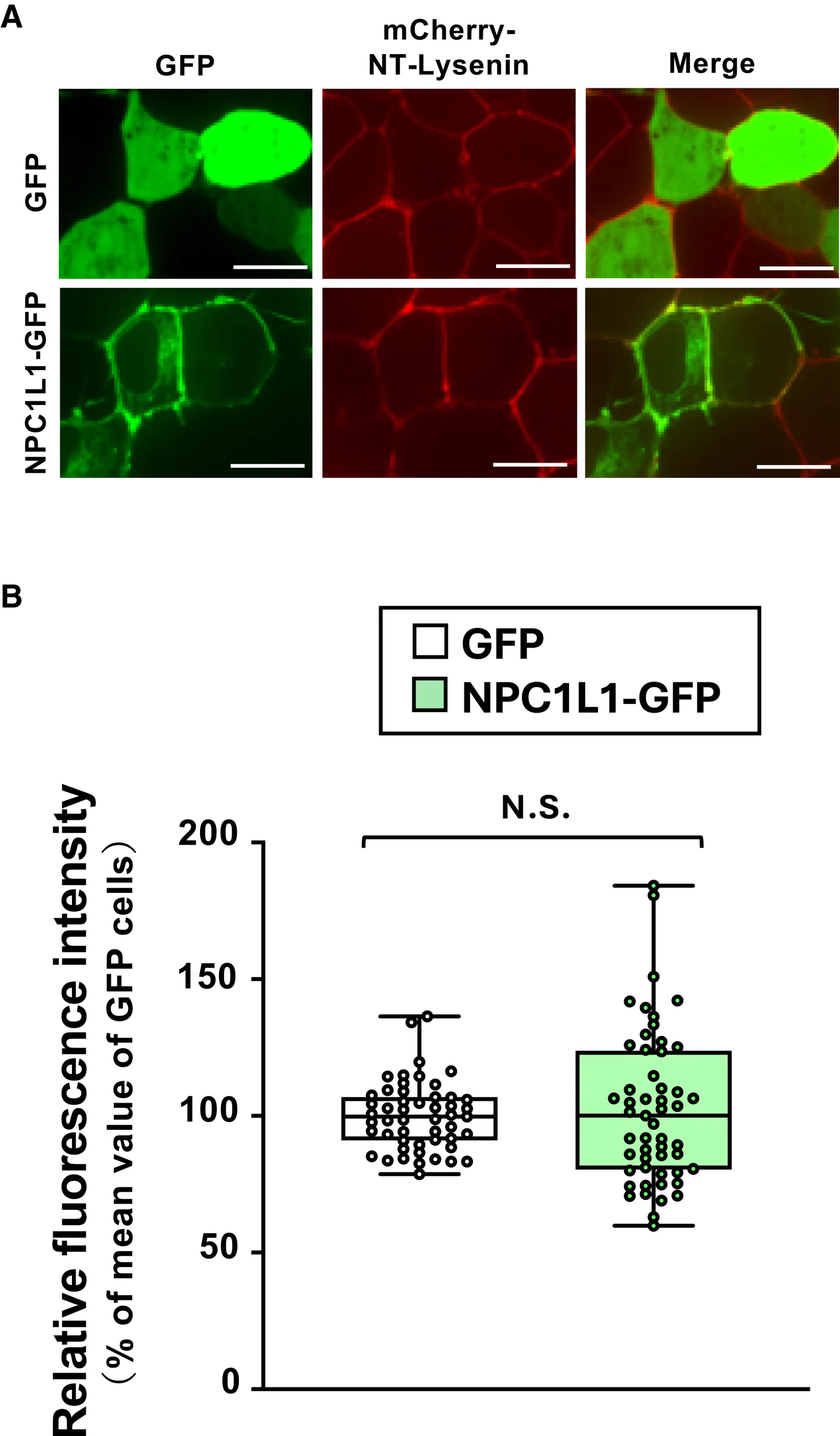
Effect of NPC1L1 on sphingomyelin distribution in outer leaflets of the plasma membrane (A) McA-RH7777 cells were transfected with either GFP-expressing vector (control) or NPC1L1-GFP-expressing vector. Forty-eight hours after transfection, the cells were labeled with recombinant mCherry-NT-lysenin probe for 15 min. Representative images of GFP fluorescence, mCherry fluorescence, and merged composites are shown. Scale bars represent 20 μm. (B) Quantification of mCherry fluorescence intensity on the PM. Relative fluorescence intensity compared with the mean value of GFP-expressing cells (GFP cells) is shown (*n* = 51 [GFP cells] and 53 [NPC1L1-GFP cells]). Box and whiskers plots represent median (line in box center), first and third quartiles (lower and upper box borders, respectively), and minimum and maximum values (whiskers). Dots represent individual data points. N.S., not significantly different using Student’s *t* test.

### NTD of NPC1L1 is barely involved in regulation of accessible cholesterol distribution across PM leaflets

Next, to investigate whether the extracellular N-terminal domain (NTD) of NPC1L1, a cholesterol-binding domain essential for NPC1L1-mediated cholesterol uptake, is involved in the regulation of accessible cholesterol distribution across PM leaflets, we evaluated the effects of a dysfunctional L216A mutation in the NTD (Figure 3A) on NPC1L1-mediated regulation of accessible cholesterol in the PM. Western blot and cell-surface biotinylation analyses revealed that total protein expression level, band pattern, and PM expression of the L216A mutant NPC1L1 (NPC1L1^L216A^) were almost identical to those of wild-type (WT) NPC1L1 (NPC1L1^WT^) in McA-RH7777 cells (Figure 3B). In addition, consistent with previous reports,^10^^,^^28^ the micellar cholesterol uptake assay demonstrated that NPC1L1^L216A^ had significantly lower cholesterol uptake activity than NPC1L1^WT^ (Figure 3C).

**Figure 3:**
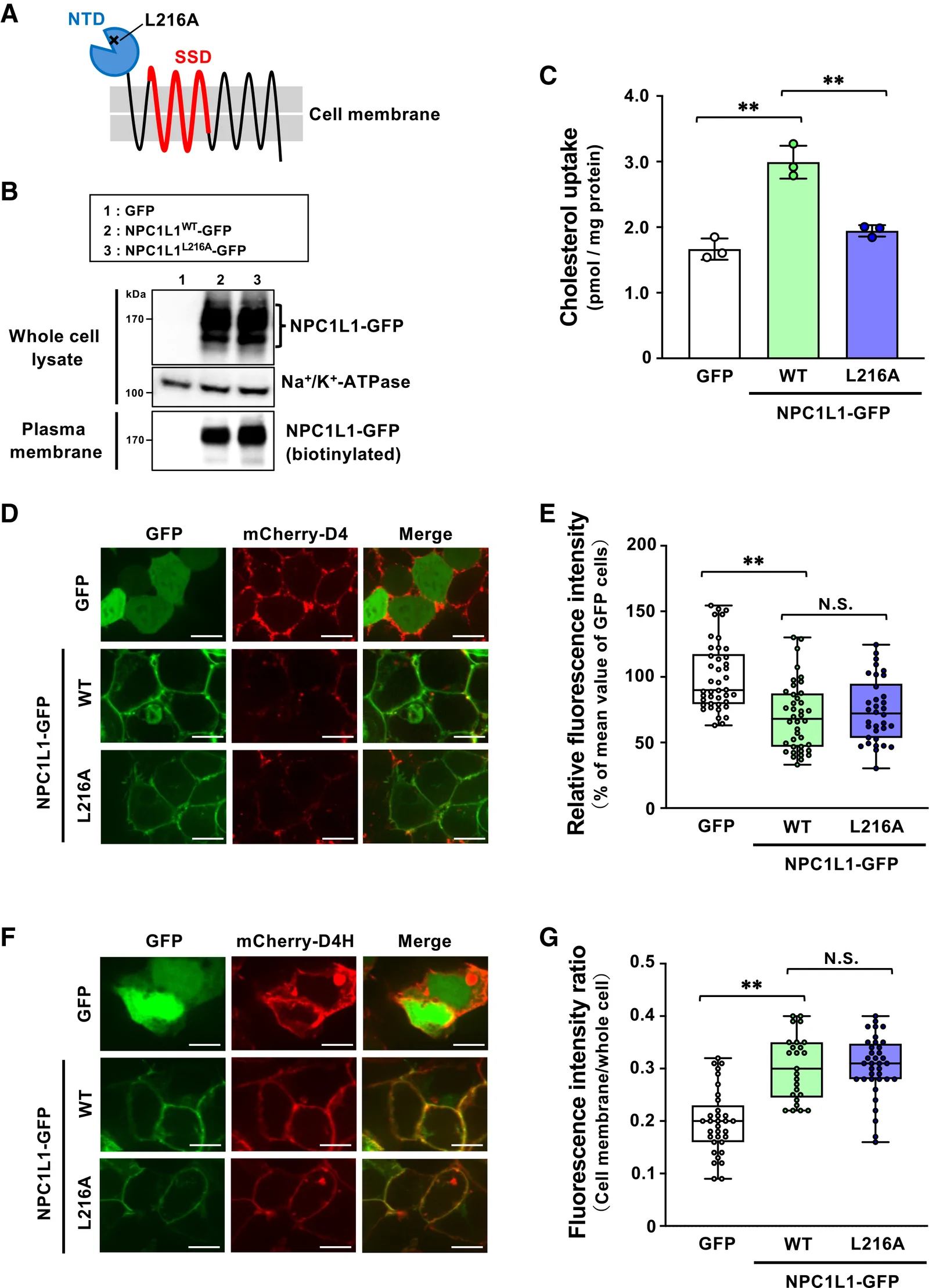
Effect of the cholesterol-binding-deficient mutation at the N-terminal domain of NPC1L1 (L216A) on regulation of accessible cholesterol distribution between lipid bilayers of the plasma membrane (A) A schematic illustration showing the N-terminal domain (NTD) and its L216A mutation in NPC1L1. (B) Total and plasma membrane expression of GFP-tagged wild-type NPC1L1 (NPC1L1^WT^-GFP) and L216A mutant NPC1L1 (NPC1L1^L216A^-GFP) in McA-RH7777 cells transfected with the indicated vectors, as determined by western blotting and cell-surface biotinylation assays. Na^+^/K^+^-ATPase was used as loading control. (C) Cholesterol uptake by McA-RH7777 cells transfected with either a GFP-expressing vector, NPC1L1^WT^-GFP-expressing vector, or NPC1L1^L216A^-GFP-expressing vector after 20-min incubation with [^3^H]cholesterol-containing micelles. Bars represent mean ± SD. Dots on the bars represent each individual data point (*n* = 3). ∗∗*p* < 0.01, significantly different between the groups using Sidak’s multiple comparison test. (D) Fluorescence microscopy images of GFP-expressing, NPC1L1^WT^-GFP-expressing, and NPC1L1^L216A^-GFP-expressing McA-RH7777 cells labeled with recombinant mCherry-D4 probe for 15 min. Representative images of GFP fluorescence, mCherry fluorescence, and merged composites are shown. Scale bars represent 20 μm. (E) Quantification of mCherry fluorescence intensity on the plasma membrane (PM). Relative fluorescence intensity compared with the mean value of GFP-expressing cells (GFP cells) is shown (*n* = 42 [GFP cells], 40 [NPC1L1^WT^-GFP cells], and 34 [NPC1L1^L216A^-GFP cells]). (F) McA-RH7777 cells were co-transfected with mCherry-D4H-expressing vector and either GFP-, NPC1L1^WT^-GFP-, or NPC1L1^L216A^-GFP-expressing vector. Representative images of GFP fluorescence, mCherry fluorescence, and merged composites taken 48 h after transfection are shown. Scale bars represent 20 μm. (G) Fluorescence intensity ratio calculated as mCherry fluorescence intensity at the PM divided by that of the whole cell is shown (*n* = 33 [GFP cells], 25 [NPC1L1^WT^-GFP cells], and 36 [NPC1L1^L216A^-GFP cells]). Box and whiskers plots represent median (line in box center), first and third quartiles (lower and upper box borders, respectively), and minimum and maximum values (whiskers). Dots represent individual data points. ∗∗*p* < 0.01, significantly different between the groups using Sidak’s multiple comparison test. N.S., not significantly different.

We then analyzed levels of accessible cholesterol in the outer leaflet of the PM of NPC1L1^L216A^-expressing cells using the mCherry-D4 probe. Similar to NPC1L1^WT^-expressing cells, NPC1L1^L216A^-expressing cells also exhibited reduced accessible cholesterol in the outer leaflet (Figures 3D and 3E). Additionally, analysis of accessible cholesterol in the inner leaflet of the PM using the mCherry-D4H probe, performed in both fixed cells (Figures 3F and 3G; Figure S7B) and live cells (Figure S11), did not show significant difference in the PM fluorescence ratio between NPC1L1^WT^- and NPC1L1^L216A^-expressing cells. Together with the observation that the whole-cell fluorescence intensity of mCherry-D4H was comparable between the groups (Figure S6B), these results suggest that, although the NTD is essential for micellar cholesterol uptake, it plays a minimal role in controlling the distribution of accessible cholesterol across PM leaflets.

### NTD of NPC1L1 inserts extracellular cholesterol into the outer leaflet of the PM

Although the NTD may be minimally involved in the regulation of accessible cholesterol distribution across PM leaflets, it is possible that this domain might play a role in insertion of extracellular cholesterol into the outer leaflet. To elucidate this role, NPC1L1^WT^-expressing cells and NPC1L1^L216A^-expressing cells were incubated with cholesterol-containing micelles, which are physiological donors of extracellular cholesterol to NPC1L1. After incubation, the NPC1L1^WT^-expressing cells showed an increase in accessible cholesterol in the outer leaflet, whereas NPC1L1^L216A^-expressing cells did not (Figures 4A and 4B). Furthermore, ezetimibe treatment partially attenuated the increase in outer-leaflet accessible cholesterol observed in NPC1L1^WT^-expressing cells (Figures 4C and 4D). These results suggest that the NTD may have the function of inserting extracellular (micellar) cholesterol into the outer leaflet of the PM and that this process is partially inhibited by ezetimibe.

**Figure 4:**
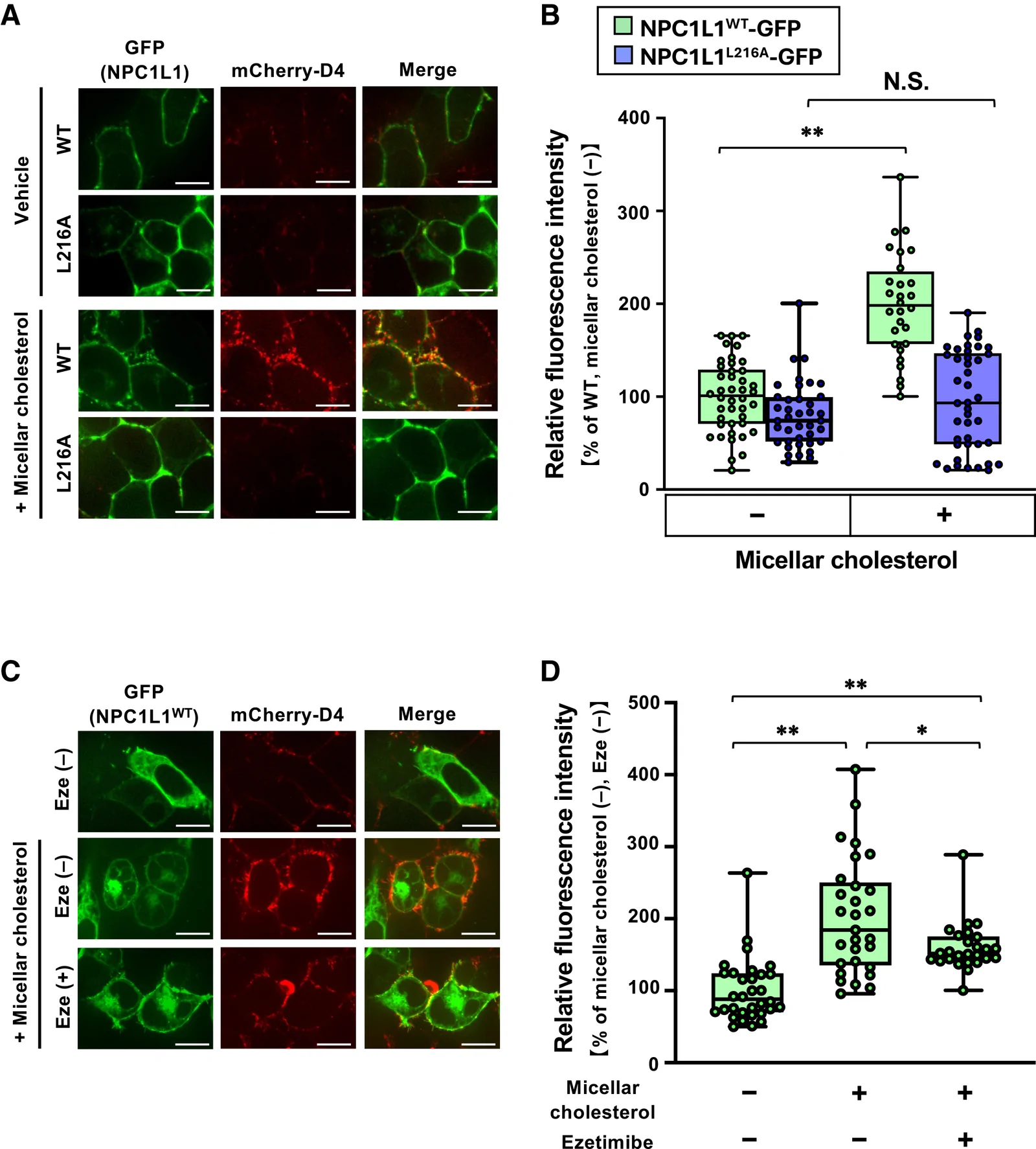
Involvement of the N-terminal domain of NPC1L1 in inserting extracellular cholesterol into the outer leaflet of the plasma membrane (A) Fluorescence microscopy images of wild-type (WT) and L216A mutant NPC1L1-GFP-expressing McA-RH7777 cells labeled with recombinant mCherry-D4 probe after 20-min preincubation in the absence (vehicle) or presence of micellar cholesterol. Representative images of GFP fluorescence, mCherry fluorescence, and merged composites are shown. Scale bars represent 20 μm. (B) Quantification of mCherry fluorescence intensity on the plasma membrane (PM). Relative fluorescence intensity compared with the mean value of WT NPC1L1-GFP-expressing cells without micellar cholesterol preincubation (WT, micellar cholesterol (−)) is shown (*n* = 42 [WT, micellar cholesterol (−)], 39 [L216A, micellar cholesterol (−)], 28 [WT, micellar cholesterol (+)], and 42 [L216A, micellar cholesterol (+)]). (C) Fluorescence microscopy images of WT NPC1L1-GFP-expressing McA-RH7777 cells treated with (+) or without (−) 30 μM ezetimibe (Eze) for 24 h, followed by a 20-min preincubation in the absence (vehicle) or presence of micellar cholesterol and subsequent labeling with recombinant mCherry-D4 probe. Representative images of GFP fluorescence, mCherry fluorescence, and merged composites are shown. Scale bars represent 20 μm. (D) Quantification of mCherry fluorescence intensity on the PM. Relative fluorescence intensity compared with the mean value of WT NPC1L1-GFP-expressing cells without ezetimibe treatment or micellar cholesterol preincubation (micellar cholesterol (−), Eze (−)) is shown (*n* = 32 [micellar cholesterol (−), Eze (−)], 29 [micellar cholesterol (+), Eze (−)], and 26 [micellar cholesterol (+), Eze (+)]). Box and whiskers plots represent median (line in box center), first and third quartiles (lower and upper box borders, respectively), and minimum and maximum values (whiskers). Dots represent individual data points. ∗*p* < 0.05, ∗∗*p* < 0.01, significantly different using Sidak’s multiple comparison test. N.S., not significantly different.

### SSD of NPC1L1 regulates accessible cholesterol distribution across PM leaflets

Besides NTD, the SSD of NPC1L1 has also been reported to play a critical role in NPC1L1-mediated cholesterol uptake.^14^ However, detailed functions of the SSD have not yet been elucidated. We hypothesized that the SSD of NPC1L1 may be involved in regulating accessible cholesterol distribution across PM leaflets. To test this hypothesis, we evaluated the effects of SSD mutations in NPC1L1 (L649R and G652A + S653A) (Figure 5A), which have been shown to reduce cholesterol uptake activity.^14^ We confirmed that protein expression levels, band patterns, and PM expression of L649R and G652A + S653A mutants were comparable to those of NPC1L1^WT^ (Figure 5B). In addition, consistent with previous reports,^14^ both the mutants exhibited significantly lower cholesterol uptake activity than NPC1L1^WT^ (Figure 5C). Next, we analyzed the distribution of accessible cholesterol in the PM of each mutant NPC1L1-expressing cell line. In the outer leaflet, both NPC1L1^L649R^ and NPC1L1^G652A+S653A^-expressing cells showed significantly stronger fluorescent intensity of mCherry-D4 compared to NPC1L1^WT^-expressing cells (Figures 5D and 5E), indicating that accessible cholesterol levels in the outer leaflet of both the mutant NPC1L1-expressing cells were higher than those of NPC1L1^WT^-expressing cells. In contrast, for the inner leaflet, although there was no significant difference in whole cell fluorescence intensity (Figure S6C), cells expressing the SSD mutants exhibited lower PM fluorescence ratios than those expressing NPC1L1^WT^ under both fixed-cell (Figures 5F and 5G; Figure S7C) and live-cell (Figure S11) conditions. These findings suggest that the SSD of NPC1L1 plays a crucial role in regulating accessible cholesterol distribution across PM leaflets and in maintaining inner-leaflet cholesterol accessibility.

**Figure 5:**
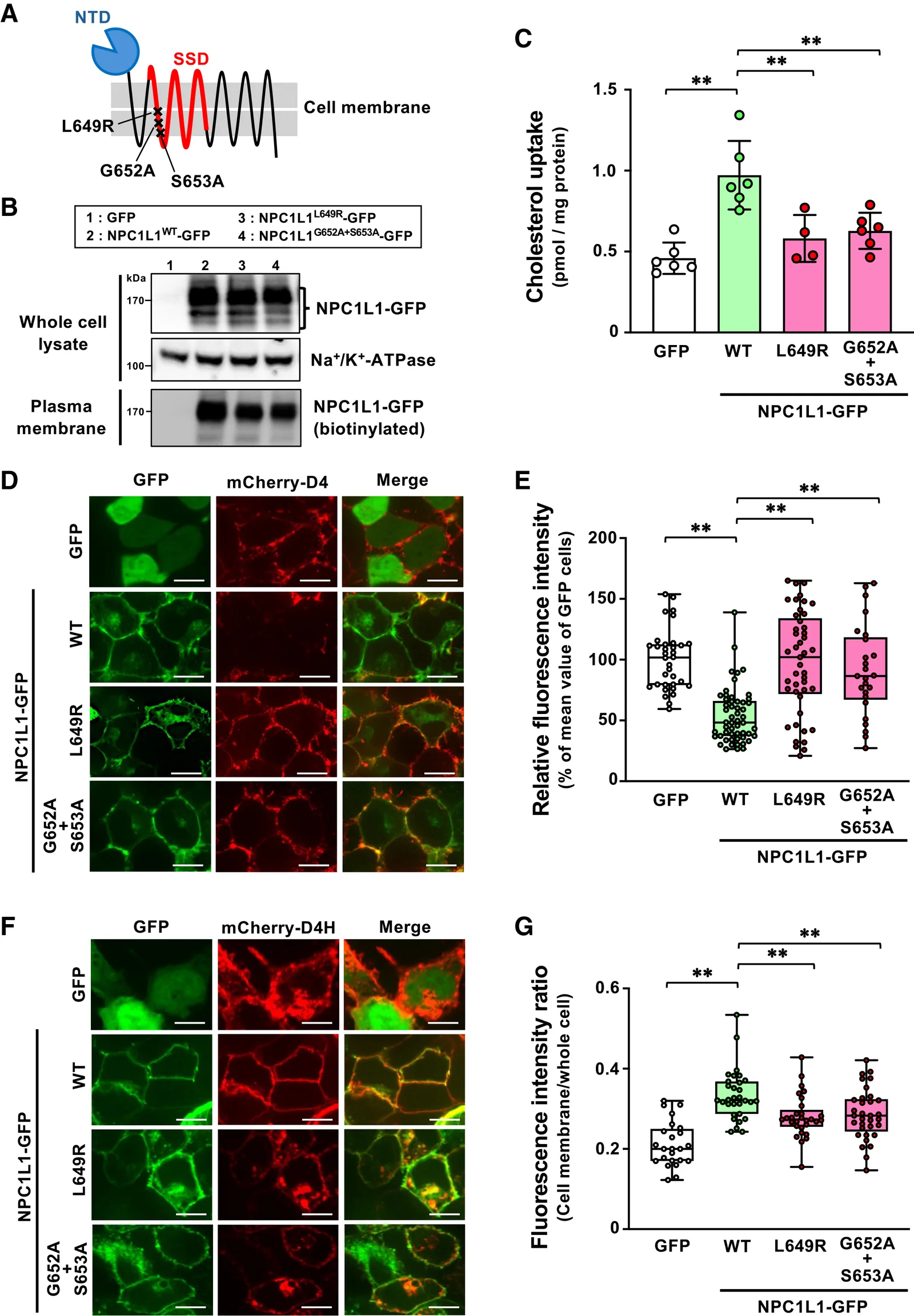
Effects of mutations at sterol-sensing domains of NPC1L1 on regulation of accessible cholesterol distribution between lipid bilayers of the plasma membrane (A) A schematic illustration showing the locations at which mutations (L649R and G652A + S653A) were introduced in the sterol-sensing domain (SSD) of NPC1L1. (B) Total and plasma membrane expression of GFP-tagged wild-type NPC1L1 (NPC1L1^WT^-GFP), L649R mutant NPC1L1 (NPC1L1^L649R^-GFP), and G652A + S653A mutant NPC1L1 (NPC1L1^G652A+S653A^-GFP) in McA-RH7777 cells transfected with the indicated vectors, as determined by western blotting and cell-surface biotinylation assays. Na^+^/K^+^-ATPase was used as loading control. (C) Cholesterol uptake by McA-RH7777 cells transfected with either GFP-expressing vector, NPC1L1^WT^-GFP-expressing vector, NPC1L1^L649R^-GFP-expressing vector, or NPC1L1^G652A+S653A^-GFP-expressing vector after 20-min incubation with [^3^H]cholesterol-containing micelles. Bars represent mean ± SD. Dots on the bars represent individual data points (n = 4–6). ∗∗*p* < 0.01, significant difference between the groups using Sidak’s multiple comparison test. (D) Fluorescence microscopy images of GFP-, NPC1L1^WT^-GFP-, NPC1L1^L649R^-GFP-, and NPC1L1^G652A+S652A^-GFP-expressing McA-RH7777 cells labeled with recombinant mCherry-D4 probe for 15 min. Representative images of GFP fluorescence, mCherry fluorescence, and merged composites are shown. Scale bars represent 20 μm. (E) Quantification of mCherry fluorescence intensity on the plasma membrane (PM). Relative fluorescence intensity compared with the mean value of GFP-expressing cells (GFP cells) is shown (*n* = 34 [GFP cells], 54 [NPC1L1^WT^-GFP cells], 45 [NPC1L1^L649R^-GFP cells], and 25 [NPC1L1^G652A+S653A^-GFP cells]). (F) McA-RH7777 cells were co-transfected with mCherry-D4H-expressing vector and either GFP-, NPC1L1^WT^-GFP-, NPC1L1^L649R^-GFP-, or NPC1L1^G652A+S653A^-GFP-expressing vector. Representative images of GFP fluorescence, mCherry fluorescence, and merged composites taken 48 h after transfection are shown. Scale bars represent 20 μm. (G) Fluorescence intensity ratio calculated as mCherry fluorescence intensity at the PM divided by that of the whole cell is shown (*n* = 24 [GFP cells], 31 [NPC1L1^WT^-GFP cells], 29 [NPC1L1^L649R^-GFP cells], and 33 [NPC1L1^G652A+S653A^-GFP cells]). Box and whiskers plots represent median (line in box center), first and third quartiles (lower and upper box borders, respectively), and minimum and maximum values (whiskers). Dots represent individual data points. ∗∗*p* < 0.01, significantly different between the groups using Sidak’s multiple comparison test.

### Vesicular endocytosis of NPC1L1 is not required for the regulation of accessible cholesterol distribution across PM leaflets

We then investigated whether vesicular endocytosis of NPC1L1 contributes to the regulation of accessible cholesterol distribution across PM leaflets. To this end, we examined a C-terminal mutant of NPC1L1 (Y1306A) (Figure 6A), which has been reported to impair cholesterol-dependent endocytosis and vesicular cholesterol uptake activity of NPC1L1.^6^ We confirmed that the protein expression levels, band patterns, and PM localization of the Y1306A mutant (NPC1L1^Y1306A^) were comparable to those of NPC1L1^WT^ (Figure 6B). Consistent with previous reports, cholesterol repletion after depletion induced internalization of NPC1L1^WT^ from the cell surface, whereas cholesterol-induced internalization of NPC1L1^Y1306A^ was markedly attenuated (Figure S12). In contrast to previous reports,^6^ however, NPC1L1^Y1306A^ exhibited ezetimibe-sensitive cholesterol uptake activity comparable to that of NPC1L1^WT^ in our experimental system (Figure 6C). These findings may reflect a relatively greater contribution of non-vesicular cholesterol uptake pathways under our experimental conditions.

**Figure 6:**
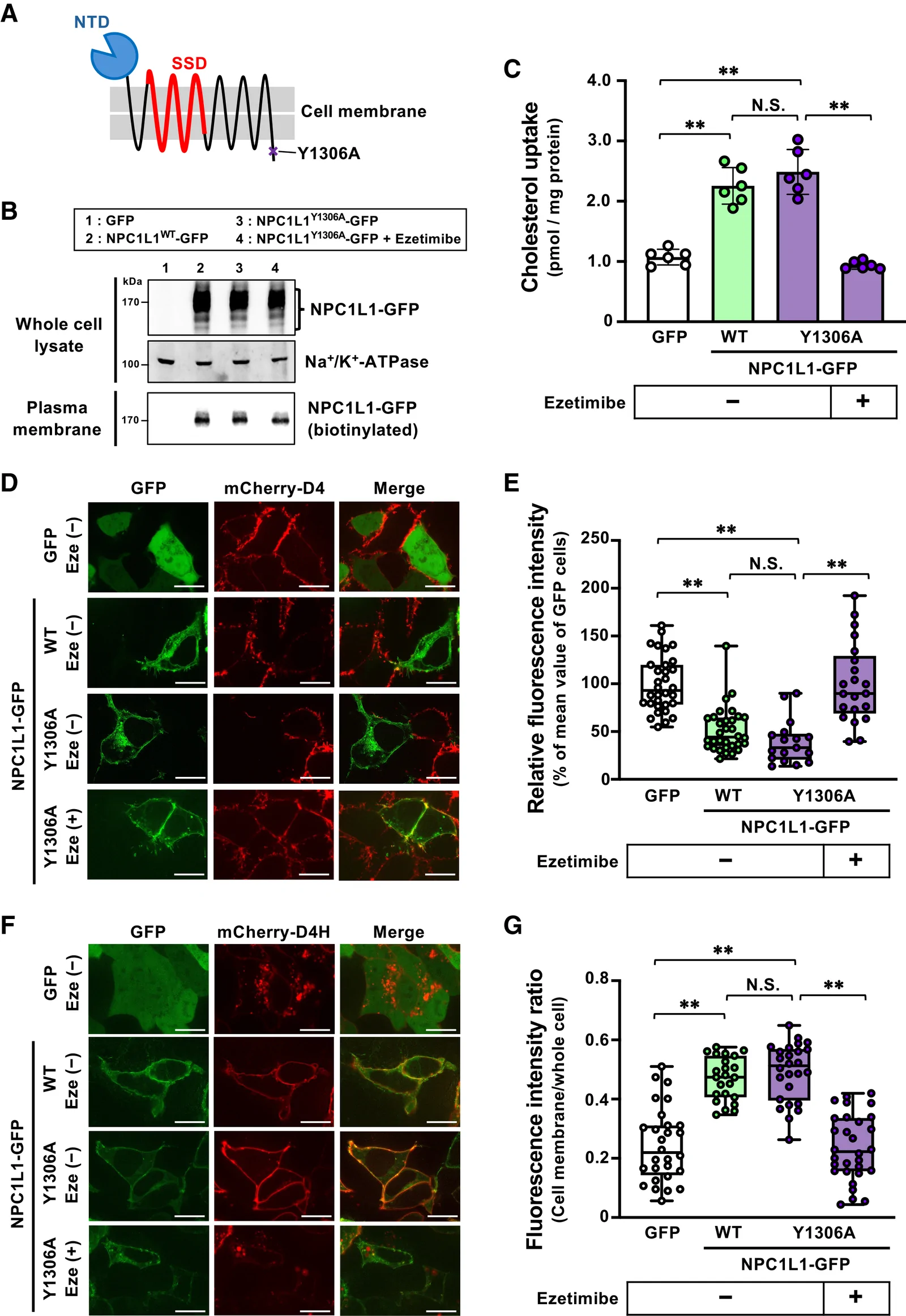
Effect of the endocytosis-impaired C-terminal NPC1L1 mutant (Y1306A) on the regulation of accessible cholesterol distribution between lipid bilayers of the plasma membrane (A) A schematic illustration showing the location of the Y1306A mutation in NPC1L1. (B) Total and plasma membrane expression of GFP-tagged wild-type NPC1L1 (NPC1L1^WT^-GFP) and Y1306A mutant NPC1L1 (NPC1L1^Y1306A^-GFP) in McA-RH7777 cells transfected with the indicated vectors and treated with or without 30 μM ezetimibe for 24 h, as determined by western blotting and cell-surface biotinylation assays. Na^+^/K^+^-ATPase was used as loading control. (C) Cholesterol uptake by McA-RH7777 cells transfected with either a GFP-expressing vector, NPC1L1^WT^-GFP-expressing vector, or NPC1L1^Y1306A^-GFP-expressing vector after a 20-min incubation with [^3^H]cholesterol-containing micelles in the absence (−) or presence (+) of 30 μM ezetimibe. Bars represent mean ± SD. Dots on the bars represent each individual data point (*n* = 6). ∗∗*p* < 0.01, significantly different between the groups using Sidak’s multiple comparison test. N.S., not significantly different. (D) Fluorescence microscopy images of GFP-expressing, NPC1L1^WT^-GFP-expressing, and NPC1L1^Y1306A^-GFP-expressing McA-RH7777 cells treated with (+) or without (−) 30 μM ezetimibe (Eze) for 24 h, followed by labeling with recombinant mCherry-D4 probe for 15 min. Representative images of GFP fluorescence, mCherry fluorescence, and merged composites are shown. Scale bars represent 20 μm. (E) Quantification of mCherry fluorescence intensity on the plasma membrane (PM). Relative fluorescence intensity compared with the mean value of GFP-expressing cells (GFP cells) is shown (*n* = 32 [GFP cells], 33 [NPC1L1^WT^-GFP cells], 17 [NPC1L1^Y1306A^-GFP cells, Eze (−)], and 21 [NPC1L1^Y1306A^-GFP cells, Eze (+)]). (F) McA-RH7777 cells were co-transfected with mCherry-D4H-expressing vector and either GFP-, NPC1L1^WT^-GFP-, or NPC1L1^Y1306A^-GFP-expressing vector. Twenty-four hours after transfection, the cells were further incubated for 24 h in the absence (−) or presence (+) of 30 μM ezetimibe (Eze). Representative images of GFP fluorescence, mCherry fluorescence, and merged composites taken 48 h after transfection are shown. Scale bars represent 20 μm. (G) Fluorescence intensity ratio calculated as mCherry fluorescence intensity at the PM divided by that of the whole cell is shown (*n* = 28 [GFP cells], 23 [NPC1L1^WT^-GFP cells], 26 [NPC1L1^Y1306A^-GFP cells, Eze (−)], and 30 [NPC1L1^Y1306A^-GFP cells, Eze (+)]). Box and whiskers plots represent median (line in box center), first and third quartiles (lower and upper box borders, respectively), and minimum and maximum values (whiskers). Dots represent individual data points. ∗∗*p* < 0.01, significantly different between the groups using Sidak’s multiple comparison test. N.S., not significantly different.

Next, we analyzed accessible cholesterol distribution in the PM of NPC1L1^Y1306A^-expressing cells. Similar to NPC1L1^WT^-expressing cells, NPC1L1^Y1306A^-expressing cells exhibited reduced outer-leaflet accessible cholesterol in an ezetimibe-sensitive manner (Figures 6D and 6E). Furthermore, analysis of inner-leaflet cholesterol accessibility using the mCherry-D4H probe demonstrated that NPC1L1^Y1306A^ expression increased the PM fluorescence ratio to a level comparable to that observed in NPC1L1^WT^-expressing cells. Consistent results were obtained under both fixed-cell (Figures 6F and 6G; Figure S7D) and live-cell (Figure S11) conditions, with no significant differences between NPC1L1^WT^- and NPC1L1^Y1306A^-expressing cells. Given that whole-cell mCherry-D4H fluorescence intensity was also comparable between the two groups (Figure S6D), these findings suggest that vesicular endocytosis of NPC1L1 is not a major determinant of accessible cholesterol distribution across PM leaflets.

### Endogenous NPC1L1 regulates accessible cholesterol distribution across PM leaflets in MDA-MB-231 cells

Finally, to elucidate whether endogenous NPC1L1 is involved in the redistribution of cholesterol from the outer to the inner leaflet of the PM, we examined membrane cholesterol distribution in MDA-MB-231 cells, a breast cancer cell line reported to endogenously express NPC1L1^29^ and allow efficient gene delivery and knockdown. We first confirmed NPC1L1 mRNA expression (Figure 7A) and protein expression (Figure 7B) in MDA-MB-231 cells. We then elucidated the effect of ezetimibe treatment on PM cholesterol distribution. Analyses using the GFP-D4 probe demonstrated that ezetimibe treatment significantly increased outer-leaflet cholesterol levels (Figures 7C and 7D). In contrast, analyses using the mCherry-D4H probe showed that ezetimibe treatment significantly decreased the PM fluorescence ratio (Figures 7E and 7F), while whole-cell fluorescence intensity was not significantly affected by ezetimibe (Figure S6E), indicating a reduction of cholesterol in the inner leaflet by ezetimibe treatment. Notably, the concentration dependence of ezetimibe was similar to that observed in NPC1L1^WT^-GFP-overexpressing McA-RH7777 cells, with effects becoming apparent at ∼2 μM and reaching near-maximal levels at ∼10 μM (Figures S3C and S3D).

**Figure 7:**
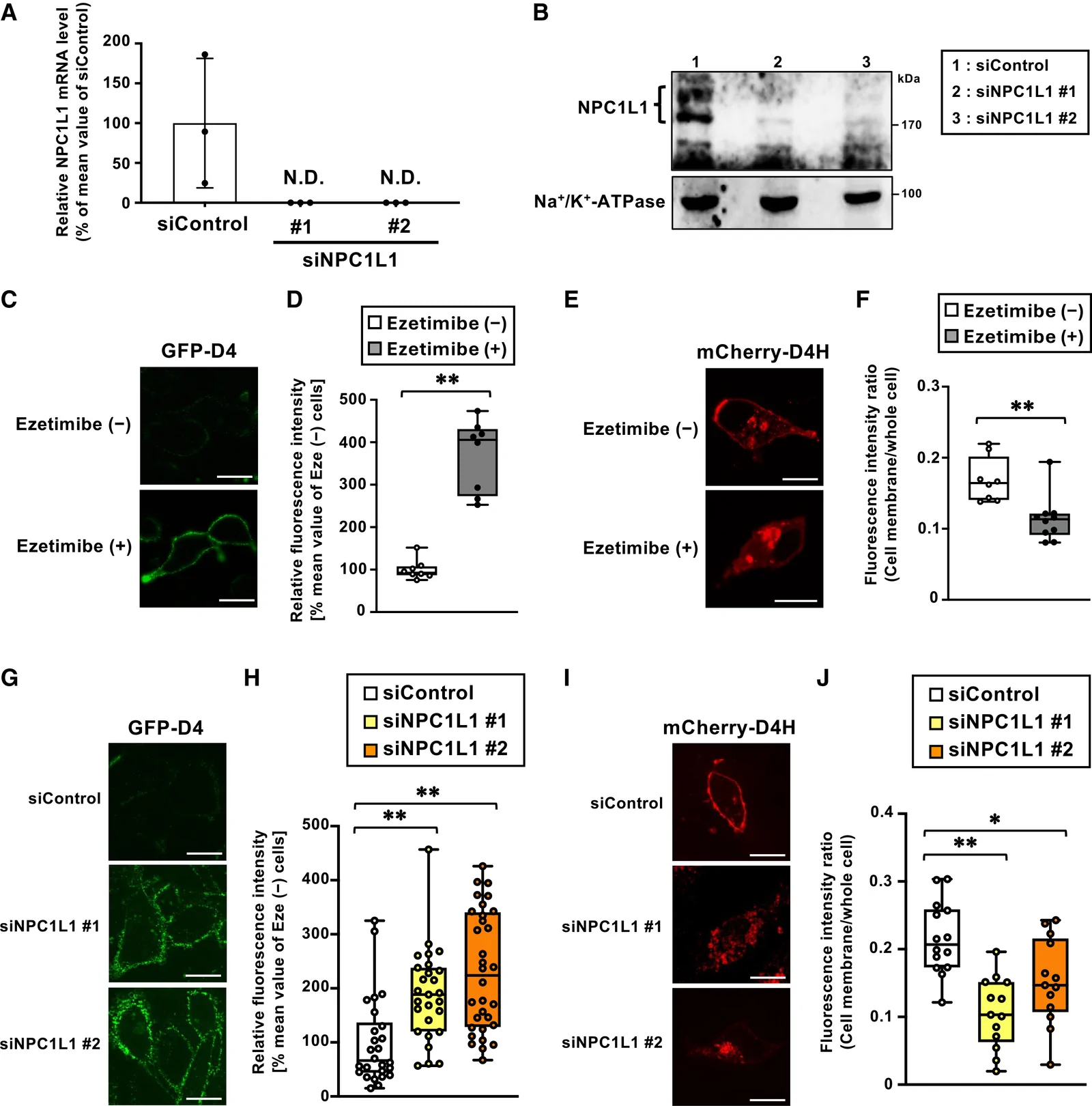
Involvement of endogenous NPC1L1 on regulation of accessible cholesterol distribution between lipid bilayers of the plasma membrane in MDA-MB-231 cells (A) Relative mRNA expression levels of MDA-MB-231 cells transfected with siControl, siNPC1L1 #1, and siNPC1L1 #2 were analyzed by real-time quantitative PCR. Bars represent mean ± SD. Dots on the bars represent individual data points (*n* = 3). N.D., not detected. (B) Protein expression of endogenous NPC1L1 in MDA-MB-231 cells transfected with siControl, siNPC1L1 #1, and siNPC1L1 #2, as determined by western blotting. Na^+^/K^+^-ATPase was used as loading control. (C) MDA-MB-231 cells were incubated for 1 h in the absence (−) or presence (+) of 40 μM ezetimibe, followed by labeling with recombinant GFP-D4 probe for 15 min. Representative images of GFP fluorescence are shown. Scale bars represent 20 μm. (D) Quantification of intensity of GFP fluorescence on the plasma membrane (PM). Relative fluorescence intensity compared with the mean value of ezetimibe-untreated cells (ezetimibe (−)) is shown (*n* = 8). (E) MDA-MB-231 cells were transfected with mCherry-D4H-expressing vector. Forty-eight hours after transfection, cells were incubated further for 1 h in the absence (−) or presence (+) of 40 μM ezetimibe. Representative images of mCherry fluorescence are shown. Scale bars represent 20 μm. (F) Fluorescence intensity ratio calculated as mCherry fluorescence intensity at the PM divided by that of the whole cell is shown (*n* = 8 [ezetimibe (−)] and 10 [ezetimibe (+)]). (G) MDA-MB-231 cells transfected with siControl, siNPC1L1 #1, and siNPC1L1 #2 were labeled with recombinant GFP-D4 probe for 15 min. Representative images of GFP fluorescence are shown. Scale bars represent 20 μm. (H) Quantification of intensity of GFP fluorescence on the plasma membrane (PM). Relative fluorescence intensity compared with the mean value of siControl cells is shown (*n* = 26 [siControl], 27 [siNPC1L1 #1], and 32 [siNPC1L1 #2]). (I) MDA-MB-231 cells were transfected with siControl, siNPC1L1 #1, and siNPC1L1 #2. Forty-eight hours after siRNA transfection, cells were transfected with mCherry-D4H-expressing vector and incubated for additional 24 h. Representative images of mCherry fluorescence are shown. Scale bars represent 20 μm. (J) Quantification of mCherry fluorescence intensity at the PM. Ratio of fluorescence intensity calculated as mCherry fluorescence intensity at the PM divided by that of the whole cell is shown (*n* = 14 [siControl], 13 [siNPC1L1 #1], and 13 [siNPC1L1 #2]). (D, F, H, and J) Box and whiskers plots represent median (line in box center), first and third quartiles (lower and upper box borders, respectively), and minimum and maximum values (whiskers). Dots represent individual data points. ∗*p* < 0.05, ∗∗*p* < 0.01, significantly different using Student’s *t* test (D and F) or Sidak’s multiple comparison test (H and J).

In addition, we conducted the same assays using NPC1L1-knockdown MDA-MB-231 cells (siNPC1L1 #1 and siNPC1L1 #2). We confirmed reduced NPC1L1 mRNA expression (Figure 7A) and protein expression (Figure 7B) in both knockdown cell lines compared with siControl cells. Consistent with the effects observed in experiments with ezetimibe treatment, both NPC1L1-knockdown cell lines exhibited a significant increase in GFP-D4 fluorescence, indicating that outer-leaflet cholesterol levels were elevated compared with siControl cells (Figures 7G and 7H). Conversely, the PM fluorescence ratio of mCherry-D4H was significantly decreased in both knockdown cell lines compared to siControl cells (Figures 7I and 7J), whereas whole cell mCherry-D4H fluorescence intensity was not significantly affected by NPC1L1 knockdown (Figure S6F). These results indicate that endogenous NPC1L1 plays a functional role in regulating the distribution of accessible cholesterol between the outer and inner leaflets of the PM in MDA-MB-231 cells.

## Discussion

This study suggests that NPC1L1 regulates the distribution of accessible cholesterol between the outer and inner leaflets of the PM (Figure 1). To the best of our knowledge, this is the first report to demonstrate that NPC1L1 not only imports extracellular cholesterol into the cell but also controls distribution of accessible cholesterol between lipid bilayers of the PM. Recently, the use of fluorescent protein- (or molecule-) tagged D4 and D4H has enabled to separately quantify accessible cholesterol levels in the outer and inner leaflets of the PM. Notably, by using this technique, cholesterol levels in the outer leaflet have been reported to be more than 10-fold higher than those in the inner leaflet of PM in several types of cultured cells, suggesting that cholesterol distribution across the lipid bilayer is not uniform.^22^ One factor contributing to this imbalance is interaction between cholesterol and phospholipids in the PM. Cholesterol forms complexes with sphingomyelin, which is abundant in the outer leaflet of the PM. Such complex formation prevents the free flip-flop (movement between lipid bilayers) of cholesterol, thereby leading to an imbalance in which the outer leaflet has more cholesterol than the inner leaflet. In our study, we found that NPC1L1 had little effect on sphingomyelin in the outer leaflet (Figure 2), suggesting that NPC1L1 is a novel factor that regulates distribution of accessible cholesterol in the lipid bilayer independent of sphingomyelin.

Besides enrichment of sphingomyelin in the outer leaflet, it has been reported that ATP-binding cassette transporter A1 (ABCA1) and ATP-binding cassette transporter G1 (ABCG1) contribute to maintain cholesterol asymmetry by promoting the redistribution of accessible cholesterol from the inner to outer leaflet of the PM.^22^^,^^30^ ABCA1 and ABCG1 are ATP-dependent cholesterol efflux transporters that export excess intracellular cholesterol to high-density lipoproteins (HDLs).^31^^,^^32^^,^^33^^,^^34^ Although these transporters have been implicated in regulating cholesterol accessibility and asymmetry across PM leaflets, no transporter that increases inner-leaflet accessible cholesterol has been reported. Our findings therefore identify NPC1L1 as a novel regulator that increases (or maintains) inner-leaflet cholesterol accessibility by controlling the distribution of accessible cholesterol across PM leaflets. Previous studies have reported inconsistent results regarding cholesterol levels between lipid bilayers.^20^^,^^21^^,^^22^^,^^23^ One possible explanation for these discrepancies may be differences in the cell-type-dependent phospholipid contents of the PM, as well as differences in the expression or activity of sterol transporters such as ABCA1, ABCG1, and NPC1L1.

We found that NTD of NPC1L1 was minimally involved in the redistribution of accessible cholesterol across PM leaflets, whereas this domain appeared to facilitate the insertion of extracellular cholesterol into the outer leaflet (Figures 3 and 4). In contrast, SSD of NPC1L1 was found to play a key role in regulating accessible cholesterol distribution across PM leaflets and increasing inner-leaflet cholesterol accessibility (Figure 5). NPC1L1-dependent cholesterol absorption has been extensively studied in the context of clathrin-mediated endocytosis and vesicular trafficking.^4^^,^^5^^,^^6^^,^^7^ However, recent findings indicate that non-vesicular cholesterol transport may also contribute to this process. Indeed, NPC1L1-mediated intestinal cholesterol absorption was recently reported to be reduced in Gramd1b and Gramd1c double KO mice, which lack the proteins involved in cholesterol transfer from the PM to the endoplasmic reticulum (ER).^12^ Given that Gramd1 proteins can access cholesterol in the inner leaflet of the PM and that cholesterol transfer to the ER is a critical process in the absorption of exogenous (dietary) cholesterol, our findings support a working model for NPC1L1-mediated non-vesicular cholesterol uptake (Figure S13). In this model, the NTD of NPC1L1 directly binds to extracellular cholesterol and inserts it into the outer leaflet of the PM, whereas the SSD of NPC1L1 increases inner-leaflet cholesterol accessibility, potentially supporting subsequent cholesterol transfer to the ER by intracellular lipid transfer proteins such as Gramd1s. Although further investigations, including studies tracking the fate of exogenous cholesterol over time, are needed, our results suggest that NPC1L1-mediated non-vesicular cholesterol uptake involves coordinated actions of two specialized functional domains that contribute to extracellular (micellar) cholesterol capture and regulation of accessible cholesterol distribution across PM leaflets in a stepwise manner.

An important consideration in interpreting our findings is that the increase in inner-leaflet accessible cholesterol observed in the present study does not necessarily indicate that cholesterol remains confined to the PM. Cholesterol associated with the inner leaflet is expected to be subsequently transferred to the ER through GRAMD1s-dependent pathways, and cholesterol reaching the ER may be rapidly esterified by acyl-CoA acyltransferase, thereby reducing the pool of accessible free cholesterol detectable by probes such as D4H and GRAM-W. Therefore, the prominent PM localization of D4H (or GRAM-W) observed in NPC1L1-expressing cells does not exclude ongoing cholesterol transport from the PM to the ER. However, because the present study was not designed to directly quantify ER cholesterol or to visualize cholesterol movement between PM leaflets, the precise kinetics and mechanism of subsequent cholesterol trafficking remain to be determined.

This study suggests that the SSD of NPC1L1 plays a role in controlling the cholesterol balance in PM lipid bilayers (Figure 5), providing novel insights into the previously unclear physiological function of the SSD. Through cryo-electron microscopy analysis, the SSD of NPC1L1 has been reported to have a tunnel structure that directly binds to cholesterol.^14^ In this study, we specifically introduced mutations into key cholesterol-binding residues—L649 and G652-S653—and found that these mutations altered accessible cholesterol distribution across PM leaflets and impaired the NPC1L1-dependent increase in inner-leaflet cholesterol accessibility (Figure 5). These functional findings are consistent with those of previous structural analyses,^14^ further supporting the notion that cholesterol recognition by the SSD is required for NPC1L1-mediated regulation of cholesterol accessibility in the PM. The SSD is a highly conserved domain found in proteins involved in maintaining cholesterol homeostasis, such as NPC1, HMG-CoA reductase, and patched 1 (PTCH1), all of which have been reported to possess cholesterol-binding structures.^35^ Notably, similar to NPC1L1, PTCH1 has been reported to decrease accessible cholesterol in the outer leaflet,^36^ indicating a conserved role of SSDs across different proteins involved in cholesterol metabolism and signaling. Further studies investigating the contribution of SSDs to cholesterol accessibility and distribution across lipid bilayers will provide important insights into the mechanisms governing cholesterol homeostasis and signaling.

Ezetimibe inhibits cholesterol absorption by targeting NPC1L1; however, its detailed mechanism of action remains incompletely understood.^13^^,^^14^ In the present study, ezetimibe treatment canceled the effect of NPC1L1 overexpression on accessible cholesterol in both the outer and inner leaflets of the PM (Figure 1; Figure S5), suggesting that ezetimibe inhibits NPC1L1-mediated regulation of accessible cholesterol distribution across PM leaflets and prevents the increase in inner-leaflet cholesterol accessibility induced by NPC1L1. In addition, ezetimibe partially attenuated the NPC1L1-dependent increase in outer-leaflet accessible cholesterol following exposure to extracellular (micellar) cholesterol, suggesting that ezetimibe may also impair the cholesterol capture and/or insertion process mediated by the NTD of NPC1L1 (Figures 4C and 4D). However, the inhibitory effect of ezetimibe on interleaflet accessible cholesterol distribution appeared more pronounced than its effect on cholesterol capture (insertion) (Figure 1), raising the possibility that disruption of SSD-dependent cholesterol regulation may play an important role in its inhibitory action. Consistent with this possibility, a recent cryo-electron microscopy analysis revealed that the ezetimibe-binding site of NPC1L1 is located within (or near) the outer leaflet of the PM and that ezetimibe binding induces conformational changes in NPC1L1, causing movement of the transmembrane regions including the SSD, which resulted in conformational changes in the cholesterol-binding site.^14^ Taken together, these findings raise the possibility that ezetimibe suppresses NPC1L1-dependent regulation of cholesterol accessibility by altering the conformation of the SSD and associated cholesterol-binding regions, thereby contributing to its inhibitory effect on NPC1L1-mediated cholesterol uptake.

Previous studies have established vesicular endocytosis-dependent mechanisms for NPC1L1-mediated cholesterol uptake, including pathways involving flotillins, lipid raft-associated proteins implicated in this process.^4^ Our findings do not contradict these models but instead suggest an additional mechanism involving SSD-dependent regulation of accessible cholesterol distribution across PM leaflets. Notably, the endocytosis-defective NPC1L1^Y1306A^ mutant produced changes in accessible cholesterol distribution comparable to those observed with NPC1L1^WT^ (Figure 6), suggesting that this process may be largely independent of the canonical NPC1L1 endocytic pathway. However, future studies will be required to determine whether flotillins or other components of the endocytic machinery contribute to the regulation of accessible cholesterol distribution and how this mechanism is integrated with established NPC1L1-mediated cholesterol uptake pathways.

Given that fluctuations in the cholesterol balance between lipid bilayers have been reported to play a crucial role in various physiological processes such as cell proliferation and signal transduction,^22^^,^^37^ our findings provide novel insights into the (patho)physiological functions of NPC1L1 beyond its well-established function of extracellular cholesterol uptake. Interestingly, NPC1L1 is expressed not only in intestinal and hepatobiliary epithelial cells^38^^,^^39^ but also in certain cancer cells, where its expression level is associated with cancer cell malignancy.^40^ Consistent with these reports, we also demonstrated that NPC1L1 is highly expressed in the breast cancer-derived MDA-MB-231 cell line and regulates the cholesterol balance between the outer and inner leaflets of the PM in an ezetimibe-sensitive manner (Figure 7). Interestingly, ezetimibe has been reported to suppress the migration and invasion of MDA-MB-231 cells,^41^ raising the possibility that this effect may be related to cellular responses triggered by alterations in membrane cholesterol distribution. Further studies are required to investigate how NPC1L1-mediated redistribution of membrane cholesterol affects cellular signaling, membrane organization, and development or progression of diseases—particularly cancer—yield valuable advances in understanding the pathophysiological significance of NPC1L1-regulated membrane cholesterol dynamics.

In addition to cholesterol, we have revealed that NPC1L1 functions as a transporter for uptake of sphingomyelin and several fat-soluble vitamins such as vitamins E and K_1_.^26^^,^^28^^,^^42^^,^^43^ It would be considerable interest to determine whether NPC1L1 similarly regulates the membrane distribution and accessibility of these substrates during their cellular uptake. However, sphingomyelin showed no significant difference in its distribution in the outer leaflet between NPC1L1-expressing and control cells (Figure 2), suggesting that the mechanisms underlying the cellular uptake of cholesterol and sphingomyelin may be different. In the future, if probes are developed that can visualize the distribution of vitamin E and vitamin K_1_, similar analyses as that for cholesterol may provide a better understanding of NPC1L1-mediated transport mechanisms of substrates other than cholesterol.

In conclusion, this study suggests that NPC1L1 regulates cholesterol accessibility across PM leaflets in an ezetimibe-sensitive manner. We further found that the NTD of NPC1L1 is involved in the capture and insertion of extracellular (micellar) cholesterol into the outer leaflet of the PM, whereas the SSD of NPC1L1 plays an important role in the subsequent regulation of accessible cholesterol distribution across PM leaflets. These findings support a model in which NPC1L1-mediated non-vesicular cholesterol uptake involves coordinated actions of two distinct functional domains that operate in a stepwise manner. Our findings contribute to a deeper understanding of the mechanisms underlying NPC1L1-mediated cholesterol transport and pharmacological actions of ezetimibe, and provide important new insights into the physiological function of the SSD, which is poorly understood.

### Limitations of the study

Several limitations of the present study should be acknowledged. The conclusions regarding NPC1L1-mediated regulation of PM cholesterol are based primarily on analyses using cholesterol probes, which detect accessible cholesterol rather than directly measuring cholesterol abundance or transbilayer cholesterol flux. Because cholesterol accessibility is influenced by multiple factors, including lipid packing, membrane composition, and protein interactions, changes in D4/D4H signals do not necessarily reflect net cholesterol movement between membrane leaflets. In addition, cholesterol undergoes relatively rapid spontaneous flip-flop across lipid bilayers; therefore, we cannot exclude the possibility that NPC1L1 affects apparent cholesterol redistribution by modulating this intrinsic process rather than directly mediating transbilayer movement. Furthermore, D4/D4H-based measurements may be influenced by probe occupancy (Figure S10) and do not provide absolute calibration of cholesterol levels. Likewise, alterations in cholesterol accessibility do not necessarily result in measurable changes in total PM cholesterol content. Although our findings suggest that NPC1L1 expression decreases outer-leaflet accessible cholesterol while increasing inner-leaflet accessible cholesterol in an SSD-dependent manner, the present data do not directly establish the underlying mechanism. Future studies employing complementary approaches capable of directly monitoring cholesterol dynamics will be required to determine whether NPC1L1 facilitates cholesterol movement between membrane leaflets or instead indirectly alters cholesterol accessibility through changes in membrane organization and lipid environment.

In addition, most mechanistic analyses in the present study were performed using NPC1L1-overexpressing McA-RH7777 cells. However, similar changes in accessible cholesterol distribution were observed in MDA-MB-231 cells expressing endogenous NPC1L1 following pharmacological inhibition or knockdown of NPC1L1 (Figure 7), suggesting that the observed effects are unlikely to be artifacts of ectopic expression. Nevertheless, because NPC1L1 is primarily expressed in intestinal epithelial cells, further validation in more physiologically relevant models, such as Caco-2 cells or intestinal organoids, will be important. Unfortunately, these analyses were not feasible in the present study because the low efficiencies of gene delivery and probe expression in these models precluded reliable D4/D4H-based imaging. Future studies using improved experimental approaches will therefore be required to confirm the physiological relevance of the proposed mechanism.

## Resource availability

### Lead contact

Requests for further information and resources should be directed to and will be fulfilled by the lead contact, Prof. Yoshihide Yamanashi (yoshihide-tky@umin.ac.jp).

### Materials availability

All common reagents used in this study are commercially available. The generated plasmids are available from the corresponding authors upon request.

### Data and code availability

• All data supporting the findings of this study are included within the article and its supplemental information or are available from the corresponding authors upon reasonable request.
• This study did not generate original code.
• Any additional information required to reanalyze the data reported in this paper is available from the corresponding authors upon request.

## Acknowledgments

This study was supported by the 10.13039/501100001691Japan Society for the Promotion of Science 10.13039/501100001691KAKENHI (grant nos. 24K22202, 24K02852, 26H01040, and 26K23629 to Y.Y.). Y.Y. has also received a research grant from the 10.13039/100007449Takeda Science Foundation.

## Author contributions

Y.Y. and T.T. designed research; Y.Y., M. Kitani, and M. Kumamaru performed research; Y.Y., M. Kitani, M. Kumamaru, T.F., and R.S. contributed new reagents/analytic tools and analyzed data; Y.Y. acquired funding; Y.Y. and T.T. supervised research; and Y.Y., M. Kitani, and T.T. wrote the paper.

## Declaration of interests

The authors declare no competing interests.

## STAR★Methods

### Key resources table

**Table:** 

REAGENT or RESOURCE	SOURCE	IDENTIFIER
**Antibodies**		

Anti-GFP rabbit IgG fraction	Thermo Fisher Scientific	Cat# A11122; RRID: AB_221569
Anti-Niemann-Pick type C1 Like-1	NOVUS Biologicals	Cat# NB400-128; RRID: AB_10000815
Na, K-ATPase antibody	Cell Signaling Technology	Cat# 3010; RRID: AB_2060983
Anti-rabbit IgG	Cytiva	Cat# NA934; RRID: AB_772206

**Chemicals, peptides, and recombinant proteins**		

Phosphatidylcholine	Nacalai Tesque	Cat# 27554–01
Oleic acid	Nacalai Tesque	Cat# 25629–75
Monoolein	Nacalai Tesque	Cat# 23408–12
NaOH solution	Nacalai Tesque	Cat# 94611–45
HCl solution	Nacalai Tesque	Cat# 37313–25
[^3^H]cholesterol (cholesterol, [1, 2, 3H(N)]-)	Perkin Elmer	Cat# NET139
Lipoprotein deficient serum (LPDS) from fetal calf	Sigma-Aldrich	–
Lysophosphatidylcholine	Santa Cruz Biotechnology	Cat# SC-473611
Sodium taurocholate	FUJIFILM Wako	Cat# 197–10033
Ezetimibe	Biosynth Carbosynth	Cat# FE23215
Imidazole	FUJIFILM Wako	Cat# 095–00015
NaH_2_PO_3_	FUJIFILM Wako	Cat# 197–09705
Na_2_HPO_3_	FUJIFILM Wako	Cat# 197–02865
Lysozyme	FUJIFILM Wako	Cat# 123–06721
NaCl	Nacalai Tesque	Cat# 31320–05
Glycerol	Nacalai Tesque	Cat# 17018–83
Isopropyl-β-thiogalactopyranoside (IPTG)	Nacalai Tesque	Cat# 19742–81
IMAC Ni-charged resin	Bio-Rad	Cat# 156–0135
Protease inhibitor cocktail	Roche	Cat# 05056489001
CBB Stain One	Nacalai Tesque	Cat# 04543–51
Paraformaldehyde	FUJIFILM Wako	Cat# 163–20145
VECTASHIELD Mounting Medium	Vector Laboratories	Cat# H-1000
Sodium dodecyl sulfate (SDS)	Nacalai Tesque	Cat# 31606–75
Tris (hydroxymethyl) aminomethane	Nacalai Tesque	Cat# 35406–91
IGEPAL CA-630	Sigma-Aldrich	Cat# 18896
Sodium deoxycholate	FUJIFILM Wako	Cat# 192–08312
Page Ruler Prestatined Protein Ladder	Thermo Fisher Scientific	Cat# 26616
ECL Prime Western Blotting Detection Reagent	Cytiva	Cat# RPN2232

**Experimental models: Cell lines**		

McA-RH7777 cells	ATCC	CRL-1601
MDA-MB-231 cells	ATCC	HTB-26

**Oligonucleotides**		

siRNA targeting sequence: NPC1L1 #1: sense: 5′-GAGGCCUUCUUAGAGGAAA-3′ antisense: 5′-UUUCCUUCUAAGAAGGCCUC-3′	This paper	N/A
siRNA targeting sequence: NPC1L1 #2: sense: 5′-GAGGCCUUCUUAGAGGAAATT-3′ antisense: 5′-UUUCCUCUAAGAAGGCCUCTT-3′	This paper	N/A
siRNA Universal Negative Control#1	Sigma-Aldrich	Cat# SIC001

**Recombinant DNA**		

pET28/His6-mCherry-D4 vector	RIKEN BRC	Cat# RDB14300
pET28/His6-EGFP-D4 vector	RIKEN BRC	Cat# RDB13961
pET28/His6-EGFP-NT-Lys vector	RIKEN BRC	Cat# RDB1348
mTagBFP2-Farnesyl-5 vector	Addgene	Cat# 55295
EGFP-GRAM-W vector	Addgene	Cat# 211701
pCold-1 vector	Takara Bio	Cat# 3361
pEGFP-N1 vector	Clontech	Cat# 6085–1

**Software and algorithms**		

Cellpose	Stringer et al.^44^	https://cellpose.readthedocs.io/en/latest/
Fiji	Schindelin et al.^45^	https://fiji.sc/
Graphpad Prism 8	Graphpad software	N/A

**Other**		

PD-10	Cytiva	Cat# 17085101

### Experimental model and study participant details

#### Cell lines

##### McA-RH7777 cells

McA-RH7777 cells were cultured in Dulbecco’s modified Eagle’s medium (DMEM) (Nacalai Tesque) supplemented with 10% fetal bovine serum (FBS) (Cosmo Bio, Tokyo, Japan), 2 mM L-glutamine (Nacalai Tesque), 1% MEM Non-Essential Amino Acid solution (NEAA) (Thermo Fisher Scientific, MA, USA), and 100 units/mL penicillin streptomycin (PCSM) (Nacalai Tesque).

##### MDA-MB-231 cells

MDA-MB-231 cells were cultured in DMEM supplemented with 10% FBS, 1% NEAA, and 100 units/mL PCSM. All cell lines were cultured at 37°C in an atmosphere supplemented with 5% CO_2_.

### Method details

#### Construction of mutated NPC1L1-GFP-expressing vectors

The expression vector for human NPC1L1 cDNA (GenBank: AY437865) with an attached GFP tag at the 3′ end (NPC1L1-GFP-expressing vector) was previously constructed by inserting human NPC1L1 cDNA into pEGFP-N1 vector (Takara Bio, Inc., Shiga, Japan).^46^ NPC1L1-GFP cDNA was mutated using the site-directed mutagenesis (SDM) technique. The following sense and antisense primers were used to construct each mutated vector. L216A (646–648 CTG>gcc) (sense primer: 5′-CAATGGTCTGGCCCCAgccGACATCACCTTCCAC-3′; antisense primer: 5′-GTGGAAGGTGATGTCggcTGGGGCCAGACCATTG-3′), L649R (1946T > g) (sense primer: 5′-ATCTCTCgGGCCCTGGGCAGCTATTCC-3′; antisense primer: 5′-CAGGGCCcGAGAGATGTACAGGAATAT-3′), G652A + S653A (1955 G > c, 1957–1958 AG > gc) (sense primer: 5′-GCCCTGGcCgcCTATTCCAGCTGGAGCCG-3′; antisense primer: 5′-GGAATAGgcGgCCAGGGCCAGAGAGATG-3′), and Y1306A (3916–3917 TA > gc) (sense primer: 5′-GACAACATCgcTGTCAACCA-3′; antisense primer: 5′-TGGTTGACAgcGATGTTGTC-3′).

#### Construction of mTagBFP2 expression vector

The mTagBFP2-Farnesyl-5 vector was modified by introducing a stop codon at the end of mTagBFP2 sequence using the site-directed mutagenesis (SDM) technique, thereby preventing translation of the downstream Farnesyl-5 sequence. The following primers were used to construct the mutated vector (sense primer: 5′-AAGCTTAATTGAGGACTCAGA-3′; antisense primer: 5′-TCTGAGTCCTCAATTAAGCTT-3′).

#### Construction of NPC1L1-mTagBFP2 expression vector

mTagBFP2 sequence from the mTagBFP2-Farnesyl-5 vector was exchanged with EGFP sequence of the NPC1L1-GFP-expressing vector at *Bam*HI/*Not* I sites using Gibson Assembly method with the following primer set (sense primer: 5′-AGTTCCGGGTACCGGATCCCATGGTGTCTAAGGGCGAA-3′; antisense primer: 5′-TCTAGAGTCGCGGCCGCTTAATTAAGCTTGTGCCCCAG-3′).

#### Preparation of micellar lipids

Reagent stock solutions were prepared by dissolving phosphatidylcholine (100 mM), lysophosphatidylcholine (20 mM), oleic acid (100 mM), monoolein (100 mM), cholesterol (10 mM), and ezetimibe (20 mM) in ethanol; and sodium taurocholate (24 mM) in DMEM. Micellar lipids dissolved in ethanol were mixed with or without (to be used as control) ezetimibe, and solvents were gently evaporated under N_2_ gas. The dried lipids were resuspended in DMEM containing sodium taurocholate and vortexed thoroughly. DMEM containing 5% LPDS was then added to adjust the final concentrations of phosphatidylcholine (35 μM), lysophosphatidylcholine (80 μM), oleic acid (0.5 mM), monoolein (0.3 mM), cholesterol (5 μM), [^3^H]cholesterol (4 nM), ezetimibe (30 μM), and sodium taurocholate (5 mM) according to experimental conditions. The prepared micelles were gently stirred at 37°C for 2 to 3 h.

#### Cholesterol uptake assay

Cholesterol uptake assay was performed as described previously.^11^ Briefly, McA-RH7777 cells were plated in collagen-coated 12-well plates at a density of 2.0 × 10^5^ cells/well. Approximately after 24 h, 2.0 μg of NPC1L1-GFP-expressing vector or GFP-expressing vector (as control) was transfected using Lipofectamine 3000 Reagent (4.0 μL of P3000 and 3.0 μL of Lipofectamine), and after another 24 h, the medium was replaced with DMEM containing 2 mM L-glutamine. The following day, the cells were pre-incubated for 1 h in DMEM containing 5% LPDS and 2 mM L-glutamine. For the ezetimibe-treated group, ezetimibe dissolved in ethanol (20 mM) was added to the pre-incubation medium to a final concentration of 30 μM. Subsequently, the cells were incubated with mixed micelles at 37°C for 20 min. Next, the cells were washed four times with ice-cold PBS containing 5 mM taurocholic acid and then solubilized in 0.2 N NaOH solution overnight at 4°C. After neutralization with 1.0 N HCl, radioactivity in the cell lysates was measured using a liquid scintillation counter. For normalization, the protein concentration in each well was determined using the BCA Protein Assay kit (Thermo Fisher Scientific).

#### Purification of his-tagged mCherry-D4, his-tagged GFP-D4, and his-tagged mCherry-Lysenin

To generate the pCold-His6-mCherry-D4 expression vector, the 6×His-mCherry-D4 sequence from pET28/His6-mCherry-D4 was ligated into pCold-1 vector at the *Eco*RI site using the Gibson Assembly method with the following primer set (sense primer: 5′-CGGTACCCTCGAGGGATCCGCCATGGTGAGCAAGGGCGAG-3′; antisense primer: 5′-ACTGCAGGTCGACAAGCTTGTTAATTGTAAGTAATACTAG-3′).

To generate the pCold-His6-EGFP-D4 expression vector, the 6×His-EGFP-D4 sequence from pET28/His6-EGFP-D4 was ligated into pCold-1 vector at the *Eco*RI site using the Gibson Assembly method with the following primer set (sense primer: 5′-CGGTACCCTCGAGGGATCCGGAGTGAGCAAGGGCGAGGAG-3′; antisense primer: 5′-ACTGCAGGTCGACAAGCTTGTTAATTGTAAGTAATACTAG-3′).

To generate the pCold-His6-mCherry-NT-Lys expression vector, the non-toxic lysenin (NT-Lys) sequence from pET28-His6-EGFP-NT-Lys was exchanged with D4 sequence of the pCold-His6-mCherry-D4 expression vector at the *Hind*III site using the Gibson Assembly method with the following primer set (sense primer: 5′-CATGGACGAGCTGTACAAGAAGCTTATAATCCTTGGAAAA-3′; antisense primer: 5′-ATCTAGACTGCAGGTCGACAAGCTTGTTAACCAACCACTT-3′).

Each constructed vector was then introduced into the *Escherichia coli* (*E. coli*) Rosetta2 strain via electroporation, and the bacteria were cultured at 37°C in Luria-Bertani (LB) medium supplemented with 50 μg/mL ampicillin and 40 μg/mL chloramphenicol for approximately 12 h with shaking. The *E. coli* culture was subsequently subcultured in fresh LB medium (containing ampicillin and chloramphenicol) and incubated with shaking at 37°C. When OD600 reached 0.7–0.8, a cold shock was induced by incubating the culture in an ice bath for approximately 30 min. IPTG was then added at a final concentration of 100 μM, and the culture was further incubated at 15°C for 20 to 22 h to induce protein expression. The bacterial cells were centrifuged and washed twice with PBS. Cell pellets thus obtained were resuspended in solubilization buffer (100 mM phosphate buffer containing 300 mM NaCl, 0.1% Triton X-100, 10% glycerol, 0.1 mg/mL lysozyme, 15 mM imidazole, and a protease inhibitor cocktail, pH 8.0) using an 18G needle. After the cells were freeze-thawed and then sonicated to disrupt them, insoluble debris was removed by centrifugation. The supernatant thus obtained was filtered, and IMAC Ni-charged resin was added and allowed to bind overnight at 4°C. The resin was then collected in a column, washed with the solubilization buffer, and subsequently washed with Triton X-100-free solubilization buffer to remove detergent. Elution buffer (100 mM phosphate buffer containing 300 mM NaCl and 300 mM imidazole, pH 8.0) was passed through the column to elute 6×His-mCherry-D4, 6×His-GFP-D4, or 6×His-mCherry-NT-Lys. Finally, the buffer was exchanged with PBS using a PD-10 desalting column, and the protein was stored at −80°C until further use.

#### Coomassie Brilliant Blue (CBB) staining

Protein concentration of the purified protein was determined using the BCA Protein Assay kit, and 5.0 μg of the sample was separated using SDS-PAGE on a 10% polyacrylamide gel. The gel was then washed with Milli-Q water, immersed in CBB Stain One, and heated in a microwave oven with occasional agitation to ensure uniform staining. After removing the staining solution, the gel was washed 2 to 3 times with Milli-Q water, heated again, and destained overnight.

#### Construction of mCherry-D4H expression vector

6×His-mCherry-D4 sequence from pET28/His6-mCherry-D4 was ligated into the pEGFP-C1 vector deleted EGFP sequence at the *Nhe* I/*Kpn* I site using Gibson Assembly method with the primer set (sense primer: 5′-GTTTAGTGAACCGTCAGATCCGACCATGGTGAGCAAGGGC-3′; antisense primer: 5′-CGGTGGATCCCGGGCCCGCGTTAATTGTAAGTAATACTAG-3′). Subsequently, Kozak sequence was inserted using SDM with the primer set (sense primer: 5′-TCCGCTAGCGCTACCGGTCGCCACCATGGTGAGCAAGGGC-3′; antisense primer: 5′-ATGGTGGCGACCGGTAGCGCTAGCGGATCTGACGGTTCAC-3′) to generate His6-mCherry-D4-expressing vector. To construct the His6-mCherry-D4H expressing vector, D434S mutation was introduced in the D4 sequence of the mCherry-D4 expression vector using SDM with the primer set (1300–1301 GA > tc) (sense primer: 5′-TTATCAAtcTAAAACAGCTCACTATTCAAC-3′; antisense primer: 5′-CTGTTTTAgaTTGATAATTTCCATCCCATG-3′).

#### Analyses using fluorescence microscopy in McA-RH7777 cells

To observe the presence of cholesterol in the outer leaflet of the cell membrane, McA-RH7777 cells (1.5 × 10^5^ cells/well) were seeded onto collagen-coated 35 mm glass bottom dishes and then cultured in complete medium (DMEM supplemented with 10% FBS, 2 mM L-glutamine, and 1% NEAA). After 24 h, 2.0 μg of NPC1L1-GFP vector, NPC1L1-BFP vector, GFP vector (pEGFP-N1 vector employed as a negative control for NPC1L1-GFP), or BFP-vector (as a negative control for NPC1L1-BFP) was transfected using Lipofectamine 3000 Reagent (4.0 μL of P3000 and 3.0 μL of Lipofectamine). The complete medium was replaced 24 h after the transfection with either a medium containing the indicated concentrations of ezetimibe (for long-term ezetimibe treatment) or control medium without ezetimibe. After additional 24 h, for the short-term ezetimibe treatment, the control medium was replaced with the medium containing 30 μM ezetimibe and the cells were incubated for 1 h. The cells were then incubated with mCherry-D4 or GFP-D4 recombinant protein (20 ng/μL, 1.0 mL) for 15 min, washed three times with PBS, and observed under a fluorescence microscope (BZ-X800; Keyence, Osaka, Japan).

To observe the presence of cholesterol in the inner leaflet of the cell membrane, McA-RH7777 cells (1.5 × 10^5^ cells/well) were seeded onto collagen-coated 35 mm glass bottom dishes and then cultured in complete medium. After 24 h, 1.0 μg of either the mCherry-D4H or EGFP-GRAM-W expression vector was co-transfected with 1.0 μg of NPC1L1-GFP vector, NPC1L1-BFP vector, GFP vector (pEGFP-N1 vector employed as a negative control for NPC1L1-GFP), or BFP-vector (as a negative control for NPC1L1-BFP) using Lipofectamine 3000 Reagent (4.0 μL of P3000 and 3.0 μL of Lipofectamine). The medium was replaced 24 h after the transfection with either complete medium containing the indicated concentrations of ezetimibe (for long-term ezetimibe treatment) or control medium without ezetimibe. After additional 24 h, for short-term ezetimibe treatment, the control medium was replaced with the medium containing 30 μM ezetimibe and the cells were incubated for 1h. For live-cell imaging, the cells were washed twice with PBS and observed under a fluorescence microscope (BZ-X800). For fixed-cell imaging, the cells were fixed by incubation with 4% paraformaldehyde at room temperature for 20 min, washed three times with PBS, and observed under a fluorescence microscope.

The captured images were segmented using Cellpose^44^ (flow threshold = 0.4, cellprob_threshold = 0.0, stitch_threshold = 0.0, model zoo: cyto), and fluorescence intensity of the whole cells was quantified using the image analysis software Fiji.^45^ The pixel size of the images was 0.15 μm/pixel. To quantify fluorescence intensity in the cell membrane region, a code was applied to reduce only the thickness of the cell membrane region to create its images, and the fluorescence intensity was measured. ErodeNum, a parameter that defines thickness of the cell membrane-associated region, was set to 10 pixels (1.50 μm) and 15 pixels (2.25 μm) to measure fluorescence intensity from the inner and outer leaflets (and their proximal regions), respectively. These values were determined based on line-scan intensity profiles of mCherry-D4-labeled and mCherry-D4H-expressing McA-RH7777 cells, which indicated that these erosion radii effectively captured membrane-associated fluorescence while minimizing contamination from cytosolic or extracellular signals. However, it should be noted that complete exclusion of cytosolic or extracellular fluorescence cannot be guaranteed in our fluorescence microscopy; therefore, the quantified fluorescence should be interpreted as signals within the membrane-proximal region rather than strictly within the plasma membrane itself.

#### Western blot analysis

McA-RH7777 cells (2.0 × 10^5^ cells/well) were cultured in 6-well plates and approximately after 24 h, 2.0 μg of each plasmid was transfected using Lipofectamine 3000 Reagent (4.0 μL of P3000 and 3.0 μL of Lipofectamine). The cells were then cultured in complete medium for an additional 48 h.

MDA-MB-231 cells (3.0 × 10^5^ cells/2 mL medium) were reverse-transfected with 30 pmol siControl (siRNA Universal Negative Control#1) (Sigma-Aldrich), siNPC1L1 #1 (sense sequence: 5′-GAGGCCUUCUUAGAGGAAA-3′, antisense sequence: 5′-UUUCCUUCUAAGAAGGCCUC-3′), or siNPC1L1 #2 (sense sequence: 5′-GAGGCCUUCUUAGAGGAAATT-3′, antisense sequence: 5′-UUUCCUCUAAGAAGGCCUCTT-3′) using 9 μL Lipofectamine RNAiMAX (Thermo Fisher Scientific), seeded into 6-well plates, and cultured in complete medium for 72 h.

The cells were washed with PBS, collected, and lysed with RIPA buffer (50 mM Tris-HCl containing 150 mM NaCl, 1% NP-40, 0.5% sodium deoxycholate, 0.1% SDS, and a protease inhibitor cocktail, pH 7.6) using a 27G needle. The lysates were centrifuged at 3,000 rpm for 10 min to collect the supernatants, which were used as samples for western blot analysis. Protein concentration of the extracted cellular proteins was measured using the BCA assay, and 20 μg was separated on an SDS-polyacrylamide gel (7%) and transferred onto a membrane at a voltage of 15V for 1 h. Subsequently, the membrane was blocked with Tris-buffered saline plus Tween 20 (TBS-T) containing 10% skim milk for 1 h at room temperature. After washing the membrane several times with TBS-T, it was incubated overnight at 4°C with 1,000-fold diluted primary antibody (anti-green fluorescent protein rabbit IgG fraction, anti-Niemann-Pick type C1 Like-1, or anti-Na, K-ATPase antibody) in TBS-T containing 1% skim milk. After washing the membrane several times with TBS-T, it was incubated for 1 h at room temperature with a 3,000-fold diluted secondary antibody (anti-rabbit IgG) in TBS-T containing 1% skim milk. Protein bands on the membrane were detected using ECL Prime Western Blotting Detection Reagent.

#### Cell-surface biotinylation assay

McA-RH7777 cells (2.0 × 10^5^ cells/well) were seeded into 6-well plates and approximately after 24 h, 2.0 μg of each plasmid was transfected using Lipofectamine 3000 Reagent (4.0 μL of P3000 and 3.0 μL of Lipofectamine) and cultured in complete medium. Forty-eight hours after the transfection, cells were washed twice with ice-cold PBS and treated with 1 mg/mL Sulfo-NHS-SS-Biotin (Pierce, Rockfold Illinois, USA) in PBS for 1 h at 4°C. Then, cells were washed with ice-cold buffer A [20 mM Tris-HCl (pH 8.0) and 150 mM NaCl] and incubated in buffer A for 15 min at room temperature. After the incubation, cells were collected by scraping and lysed in RIPA buffer. Each sample of cell lysate was incubated with 100 μL of 50% (v/v) Magnabind Streptavidin Beads (Pierce) and rotated overnight at 4°C. The avidin beads were then washed three times with RIPA buffer. Biotinylated proteins binding to the beads were eluted by incubation with SDS loading buffer containing dithiothreitol at 37°C for 30 min. The incubated mixtures were centrifugated briefly and the supernatants were subjected to western blot analysis.

#### Real-time quantitative PCR

MDA-MB-231 cells (2.0 × 10^5^ cells/well) were reverse-transfected by seeding them with 20 pmol siControl, siNPC1L1 #1, or siNPC1L1 #2 and 6 μL Lipofectamine RNAiMAX into 12-well plates and cultured in complete medium. After 72 h, total RNA was extracted from the cells with RNAiso Plus (500 μL/well) (TaKaRa, Osaka, Japan) according to the manufacturer’s instructions. The extracted total RNA was reverse-transcribed using ReverTra Ace qPCR RT Master Mix (TOYOBO, Osaka, Japan). qPCR was performed using THUNDERBIRD Next SYBR qPCR Mix (TOYOBO) and the LightCycler 96 system (Roche) at 95°C for 30 s followed by 40 cycles at 95°C for 1 s and 60°C for 30 s. Primers for human NPC1L1 (sense primer: 5′-GGTATCACTGGAAGCGAGTC-3′, antisense primer: 5′-AGGTAGAAGGTGGAGTCGAG-3′) and human β-actin (sense primer: 5′-CTTCCTTCCTGGGCATGGAGTC-3′, antisense primer: 5′-CCACACGGAGTACTTGCGCT-3′) were used.

#### Analyses using fluorescence microscopy in MDA-MB-231 cells

To observe the effect of ezetimibe on cholesterol distribution in the outer leaflet of the cell membrane, MDA-MB-231 cells (3.0 × 10^5^ cells/well) were seeded onto collagen-coated 35 mm glass bottom dishes and cultured for 48 h in complete medium. The cells were then incubated for 1 h in medium containing either vehicle (control) or 40 μM ezetimibe. Following treatment, GFP-D4 recombinant protein (20 ng/μL, 1.0 mL) was added to the cells and incubated for 15 min. The cells were washed three times with PBS and observed under a fluorescence microscope. For NPC1L1 knockdown experiments, MDA-MB-231 cells (3.0 × 10^5^ cells/well) were reverse-transfected by seeding them onto collagen-coated 35 mm glass bottom dishes together with 30 pmol siControl, siNPC1L1 #1, or siNPC1L1 #2 and 9 μL Lipofectamine RNAiMax, and then cultured in complete medium. After 72 h, GFP-D4 recombinant protein (20 ng/μL, 1.0 mL) was added and incubated for 15 min, followed by three times washes with PBS. The cells were then examined under a fluorescence microscope.

To observe the effect of ezetimibe on cholesterol distribution in the inner leaflet of the cell membrane, MDA-MB-231 cells (3.0 × 10^5^ cells/well) were seeded onto collagen-coated 35 mm glass bottom dishes and cultured in complete medium. After 24 h, 2.5 μg of mCherry-D4H expression vector was transfected using 7.5 μL FuGene HD transfection Reagent (Promega, Madison, WI, USA), and the cells were cultured for an additional 24 h in complete medium. The cells were then incubated for 1 h in medium containing either vehicle (control) or 40 μM ezetimibe, washed twice with PBS, and subsequently observed under a fluorescence microscope. For NPC1L1 knockdown experiments, MDA-MB-231 cells (3.0 × 10^5^ cells/well) were reverse-transfected by seeding them with 30 pmol siControl, siNPC1L1 #1, or siNPC1L1 #2 and 9 μL Lipofectamine RNAiMAX onto collagen-coated 35 mm glass bottom dishes, and then cultured in complete medium. After 48 h, the cells were additionally transfected with 2.5 μg of mCherry-D4H expression vector using 7.5 μL FuGene HD transfection reagent. After 24 h, the cells were washed twice with PBS and observed under a fluorescence microscope.

Quantification analyses of images were conducted as described above for McA-RH7777 cells.

### Quantification and Statistical analysis

Statistical analyses were performed using Graphpad Prism version 8. Data are presented as mean ± S.D. in Bar graphs or median (line in box center), first and third quartile (lower and upper box border, respectively), and minimum and maximum values (whiskers) in Box and whiskers plots. Data were obtained from independent biological replicates, each assayed in duplicate. The exact number of cells analyzed (n) is provided in the corresponding figure legends. Statistical significance was evaluated using Student’s *t* test for two-group comparisons, or Sidak’s multiple comparison test for multiple comparisons. Differences with *p* < 0.05 were considered statistically significant.
