# Supplementary material for: Dual functions of NPC1L1 for cholesterol transport: Cholesterol capture and regulation of inner-leaflet cholesterol accessibility
Source: iScience. 2026 Sep 24;29(10):117625. doi: 10.1016/j.isci.2026.117625 (PMC13634356; doi:10.1016/j.isci.2026.117625)
Supplement: Supplementary file 1 [file mmc1.pdf]

iScience, Volume *29*

Supplemental information

Dual functions of NPC1L1 for cholesterol transport: Cholesterol capture and regulation of inner-leaflet cholesterol accessibility

Yoshihide Yamanashi, Maiko Kitani, Megumi Kumamaru, Tomoki Fukaya, Rina Sugita, and Tappei Takada

Supplemental figures

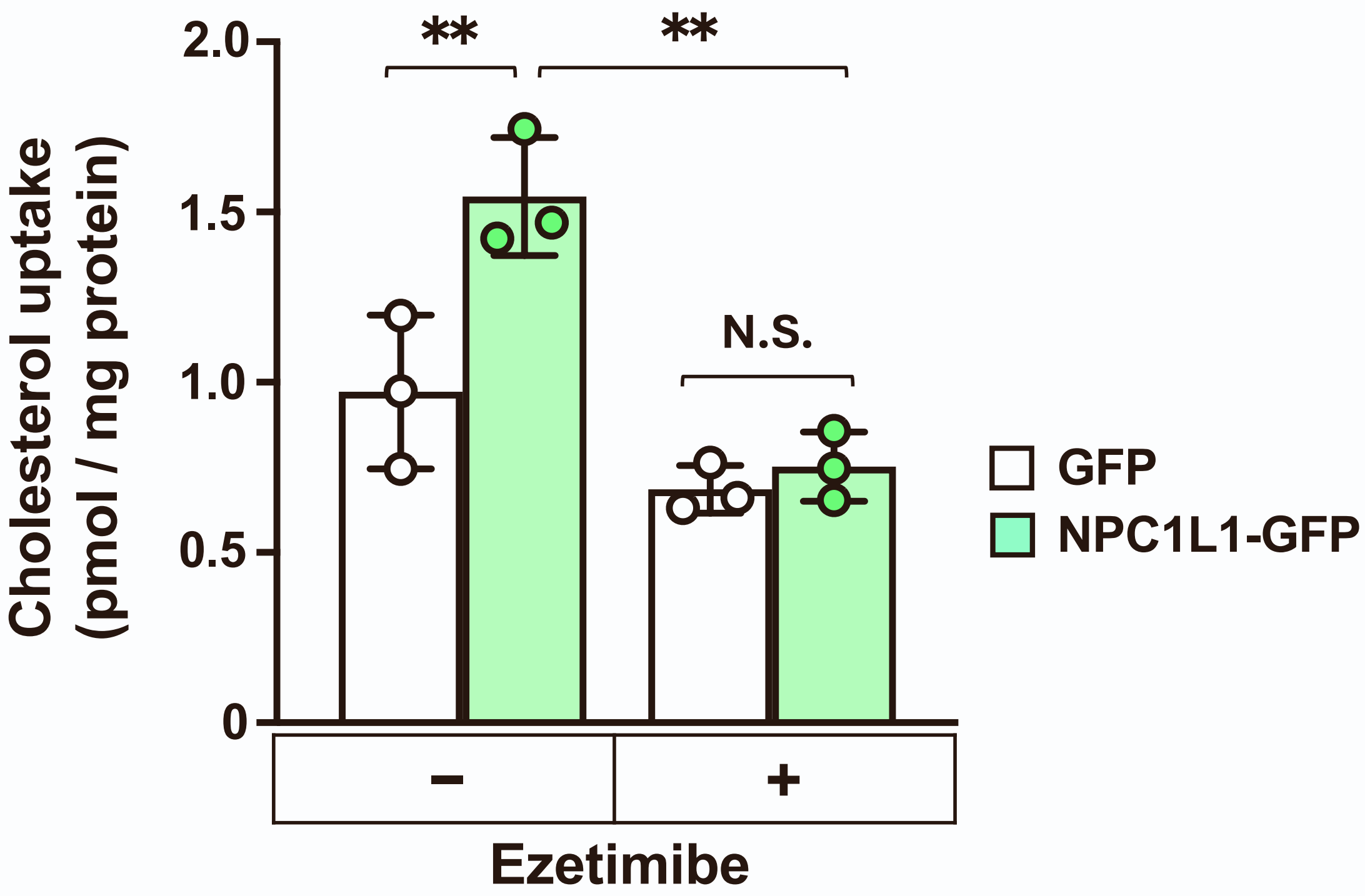

Figure S1. NPC1L1-mediated cholesterol uptake in McA-RH7777 cells. Cholesterol uptake by McA-RH7777 cells transfected with either the GFP-expressing vector (control) or the NPC1L1-GFP-expressing vector after 20-minute incubation with [^{3}H]cholesterol-containing micelles in the absence (−) or presence (+) of 30 μM ezetimibe. Bars represent mean ± S.D. Dots on the bars represent each individual data point (n=3). **p<0.01, significantly different using Sidak's multiple comparison test. N.S.; not significantly different.

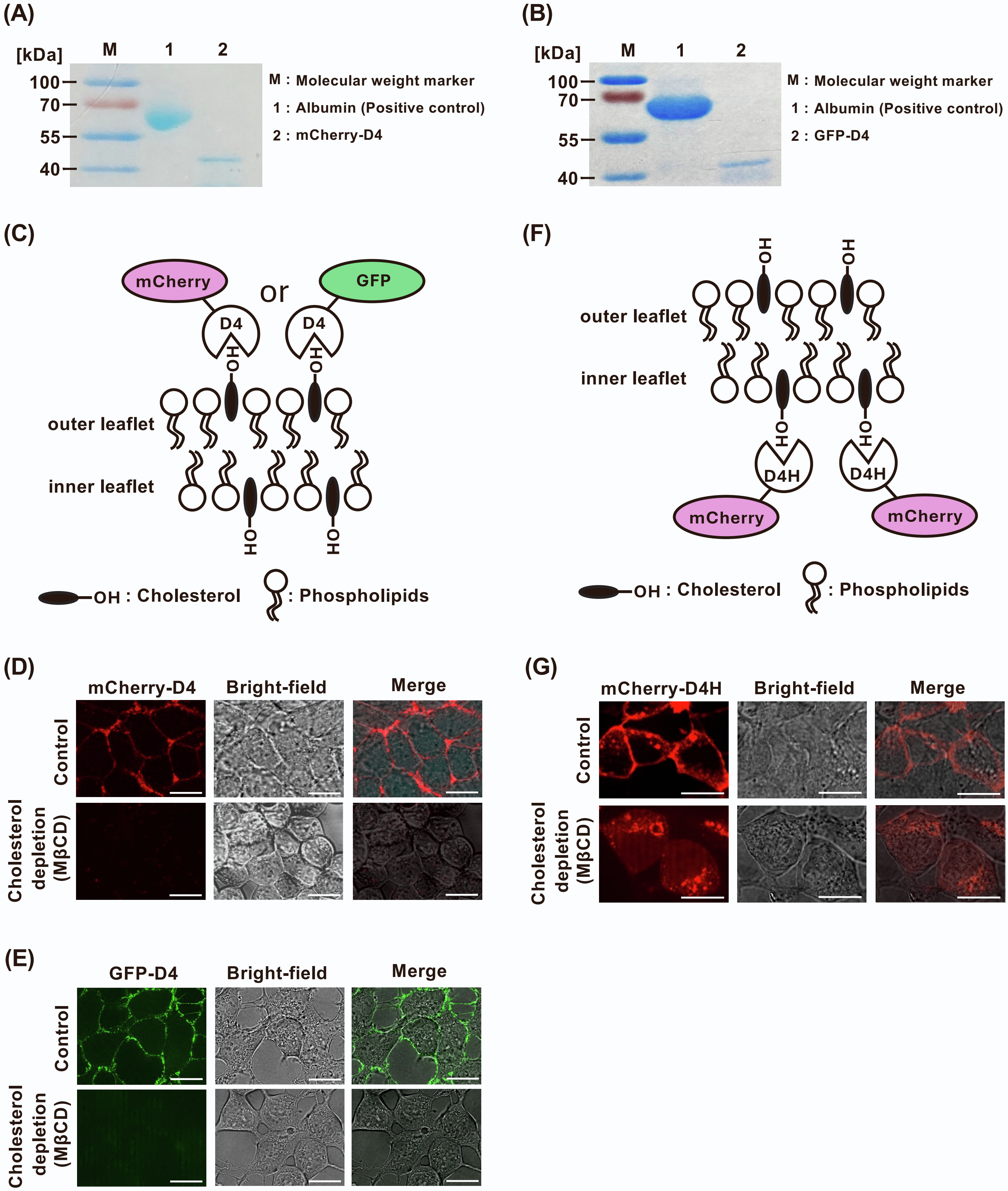

Figure S2. Detection of accessible cholesterol in the plasma membrane. (A and B) Coomassie Brilliant Blue (CBB) staining of bovine serum albumin (control) and recombinant (A) mCherry-D4 probe or (B) GFP-D4 probe after purification using Ni-charged resin. (C) A schematic illustration of the detection system for accessible cholesterol in the outer leaflet of the plasma membrane (PM) by the recombinant mCherry-D4 probe or GFP-D4 probe. (D and E) Fluorescence microscopy images of intact (control) and cholesterol-depleted (treated with 1.5% MβCD for 20 min) McA-RH7777 cells labeled with the recombinant (D) mCherry-D4 probe or (E) GFP-D4 probe. Representative images of mCherry or GFP fluorescence, bright-field, and merged composites are shown. Scale bars represent 20 μm. (F) A schematic illustration of the detection system for accessible cholesterol in the inner leaflet of the PM by the mCherry-D4H probe expressed by the mChery-D4H vector. (G) Fluorescence microscopy images of intact (control) and cholesterol-depleted (treated with 1.5% MβCD for 20 min) McA-RH7777 cells transfected with the mCherry-D4H-expressing vector. Representative images of mCherry fluorescence, bright-field, and merged composites are shown. Scale bars represent 20 μm.

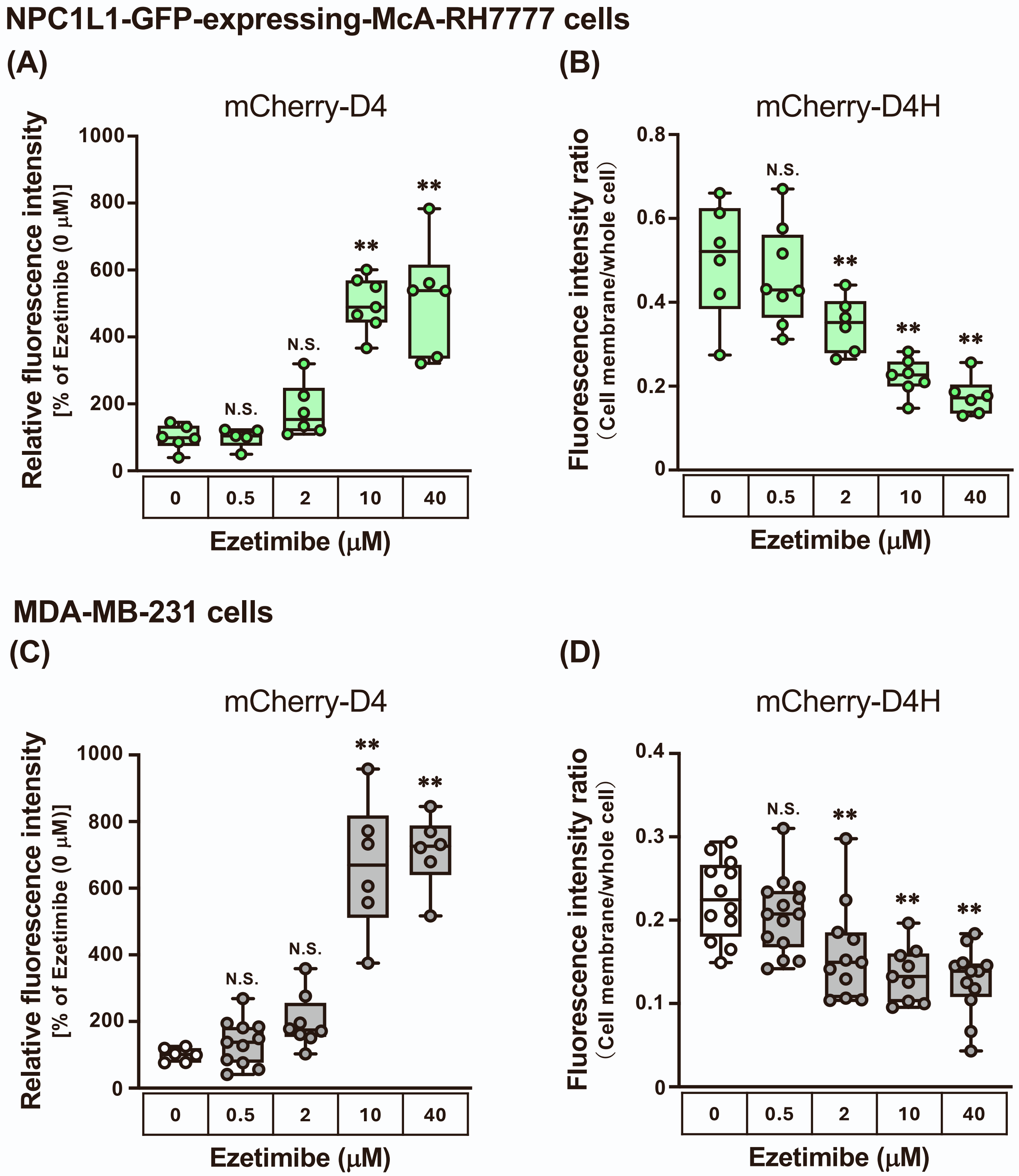

Figure S3. Concentration-dependent effects of ezetimibe on accessible cholesterol distribution across plasma membrane leaflets. (A) McA-RH7777 cells were transfected with the NPC1L1-GFP-expressing vector. Forty-eight hours after transfection, the cells were incubated for additional 24 h in medium containing the indicated concentrations of ezetimibe, followed by labeling with the recombinant mCherry-D4 probe for 15 min. Quantification of intensity of mCherry fluorescence on the plasma membrane (PM). Relative fluorescence intensity compared with the mean value of ezetimibe-untreated cells [Ezetimibe (0 μM)] is shown (n=5-7). (B) McA-RH7777 cells were co-transfected with mCherry-D4H-expressing vector and NPC1L1-GFP-expressing vector. Forty-eight hours after transfection, the cells were further incubated for 24 h in medium containing the indicated concentrations of ezetimibe. Fluorescence intensity ratio calculated as mCherry fluorescence intensity at the PM divided by that of the whole cell is shown (n=6-8). (C) MDA-MB-231 cells were incubated for 1 h in medium containing the indicated concentrations of ezetimibe, followed by labeling with recombinant GFP-D4 probe for 15 min. Quantification of intensity of GFP fluorescence on the PM. Relative fluorescence intensity compared with the mean value of ezetimibe-untreated cells [Ezetimibe (0 μM)] is shown (n=6-11). (D) MDA-MB-231 cells were transfected with mCherry-D4H-expressing vector. Forty-eight hours after transfection, cells were incubated further for 1 h in medium containing the indicated concentrations of ezetimibe. Fluorescence intensity ratio calculated as mCherry fluorescence intensity at the PM divided by that of the whole cell is shown (n=9-14). Box and whiskers plots represent median (line in box center), first and third quartile (lower and upper box border, respectively), and minimum and maximum values (whiskers). Dots represent individual data points. *$p<0.05$, **$p<0.01$, significantly different from ezetimibe-untreated cells as determined by Sidak's multiple comparison test. N.S., not significantly different.

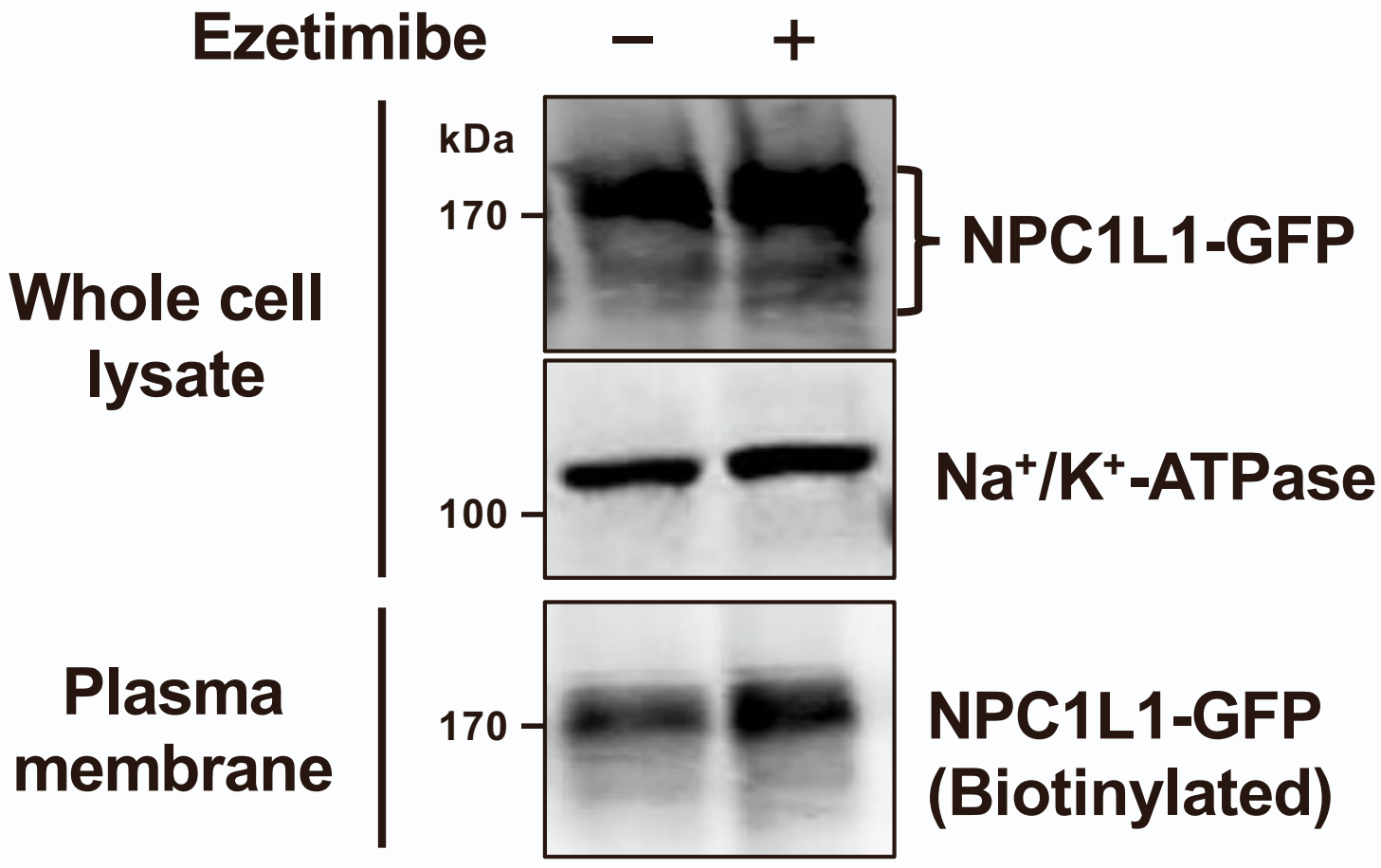

Figure S4. Effects of ezetimibe on total and plasma membrane expression of NPC1L1 in McA-RH7777 cells. Plasma membrane expression of NPC1L1-GFP was determined by a cell-surface biotinylation assay. Whole cell lysates and biotinylated proteins were prepared from McA-RH7777 cells transfected with the NPC1L1-GFP-expressing vector and incubated for 24 h in the absence (−) or presence (+) of 30 μM ezetimibe. Proteins were separated on 7% sodium dodecyl sulfate-polyacrylamide gels, transferred to membranes, and detected with anti-GFP antibody and anti-Na^+/K^+-ATPase antibody.

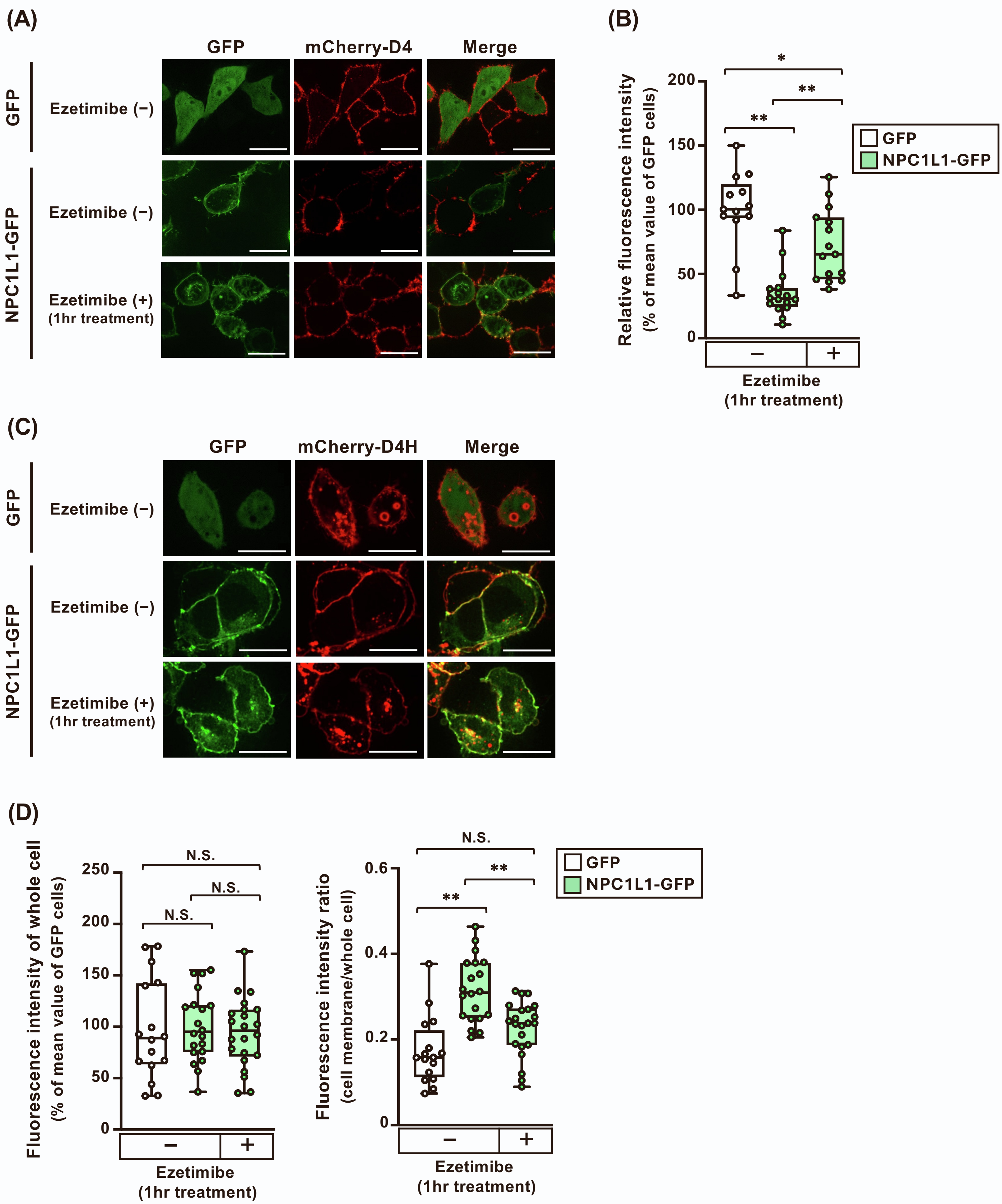

Figure S5. Effect of short-term ezetimibe treatment on NPC1L1-mediated regulation of accessible cholesterol distribution between lipid bilayers of the plasma membrane. (A) McA-RH7777 cells were transfected with either GFP-expressing vector (control) or NPC1L1-GFP-expressing vector. Forty-eight hours after transfection, the cells were incubated for additional 1 h in the absence (−) or presence (+) of 30 μM ezetimibe, followed by labeling with the recombinant mCherry-D4 probe for 15 min. Representative images of GFP fluorescence, mCherry fluorescence, and merged composites are shown. Scale bars represent 20 μm. (B) Quantification of intensity of mCherry fluorescence on the plasma membrane (PM). Relative fluorescence intensity compared with the mean value of ezetimibe-untreated GFP-expressing cells [Eze (–), GFP cells] is shown (n=13-16). (C) McA-RH7777 cells were co-transfected with mCherry-D4H-expressing vector and either GFP- or NPC1L1-GFP-expressing vector.

Forty-eight hours after transfection, the cells were further incubated for 1 h in the absence (−) or presence (+) of 30 μM ezetimibe. Representative images of GFP fluorescence, mCherry fluorescence, and merged composites are shown. Scale bars represent 20 μm. (D) Quantification of intensity of mCherry fluorescence in the whole cell and at the PM (n=16-22). Relative whole cell fluorescence intensity compared with the mean value of GFP cells is shown (left panel). Ratio of fluorescence intensity calculated as mCherry fluorescence intensity at the PM divided by that of the whole cell is shown (right panel). Box and whiskers plots represent median (line in box center), first and third quartile (lower and upper box border, respectively), and minimum and maximum values (whiskers). Dots represent individual data points. *$p<0.05$, **$p<0.01$, significantly different using Sidak's multiple comparison test. N.S., not significantly different.

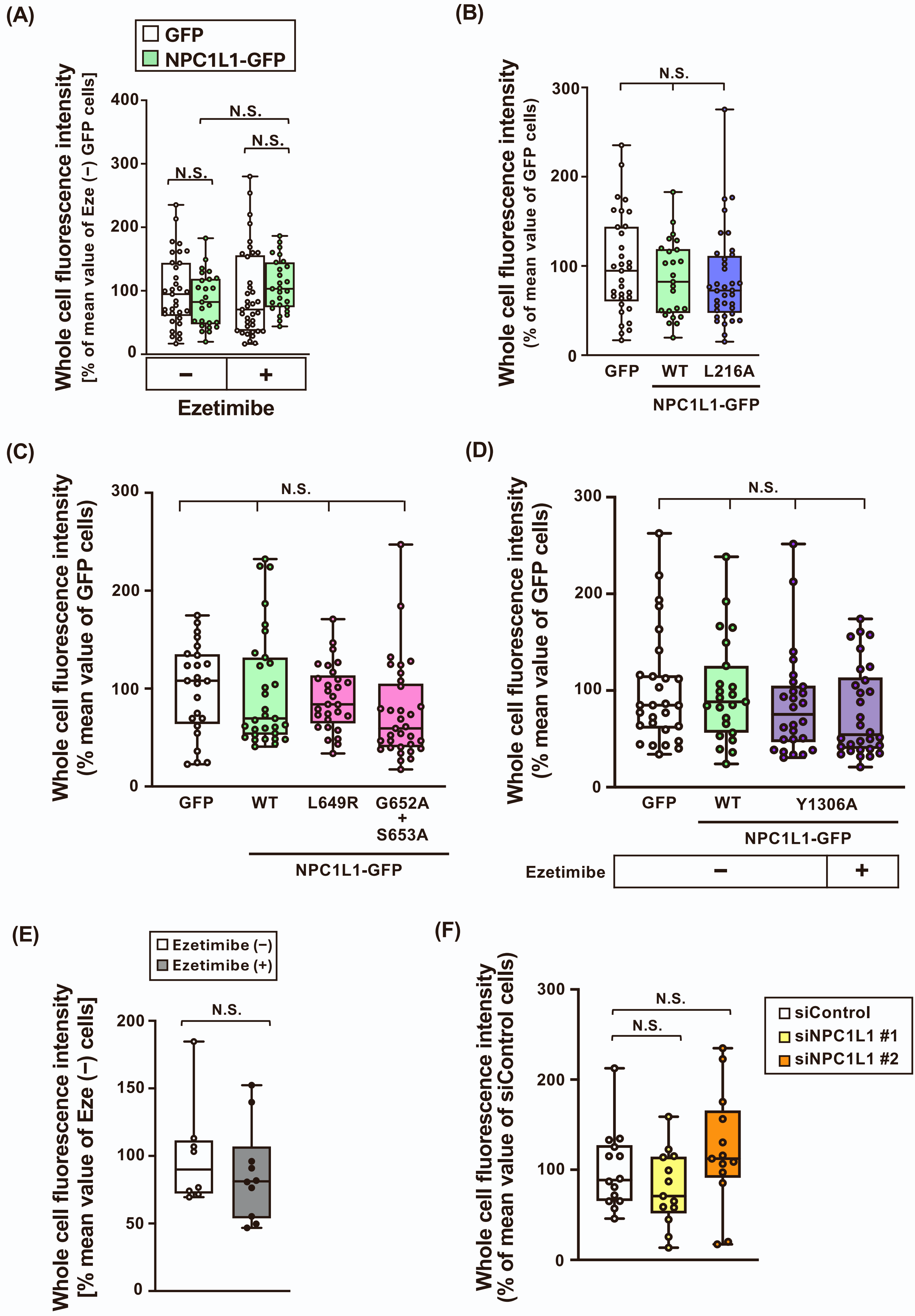

(A)
GFP
NPC1L1-GFP
Whole cell fluorescence intensity
[% of mean value of Eze (−) GFP cells]
400
300
200
100
0
N.S.
N.S.
N.S.
−
+
Ezetimibe
(B)
Whole cell fluorescence intensity
(% of mean value of GFP cells)
300
200
100
0
N.S.
GFP
WT
L216A
NPC1L1-GFP
(C)
Whole cell fluorescence intensity
(% mean value of GFP cells)
300
200
100
0
N.S.
GFP
WT
L649R
G652A
+
S653A
NPC1L1-GFP
(D)
Whole cell fluorescence intensity
(% mean value of GFP cells)
300
200
100
0
N.S.
GFP
WT
Y1306A
NPC1L1-GFP
Ezetimibe
−
+
(E)
Ezetimibe (−)
Ezetimibe (+)
Whole cell fluorescence intensity
[% mean value of Eze (−) cells]
200
150
100
50
0
N.S.
(F)
Whole cell fluorescence intensity
(% of mean value of siControl cells)
300
200
100
0
N.S.
N.S.
siControl
siNPC1L1 #1
siNPC1L1 #2

Figure S6. **Whole cell fluorescence intensity of mCherry-D4H.** Whole cell fluorescence intensity in (A) Figure 1D, (B) Figure 3G, (C) Figure 5G, (D) Figure 6G, (E) Figure 7F, and (F) Figure 7J. Box and whiskers plots represent median (line in box center), first and third quartile (lower and upper box border, respectively), and minimum and maximum values (whiskers). Dots represent individual data points. N.S., not significantly different by Student's t-test (E) or Sidak's multiple comparison test (A-D and F).

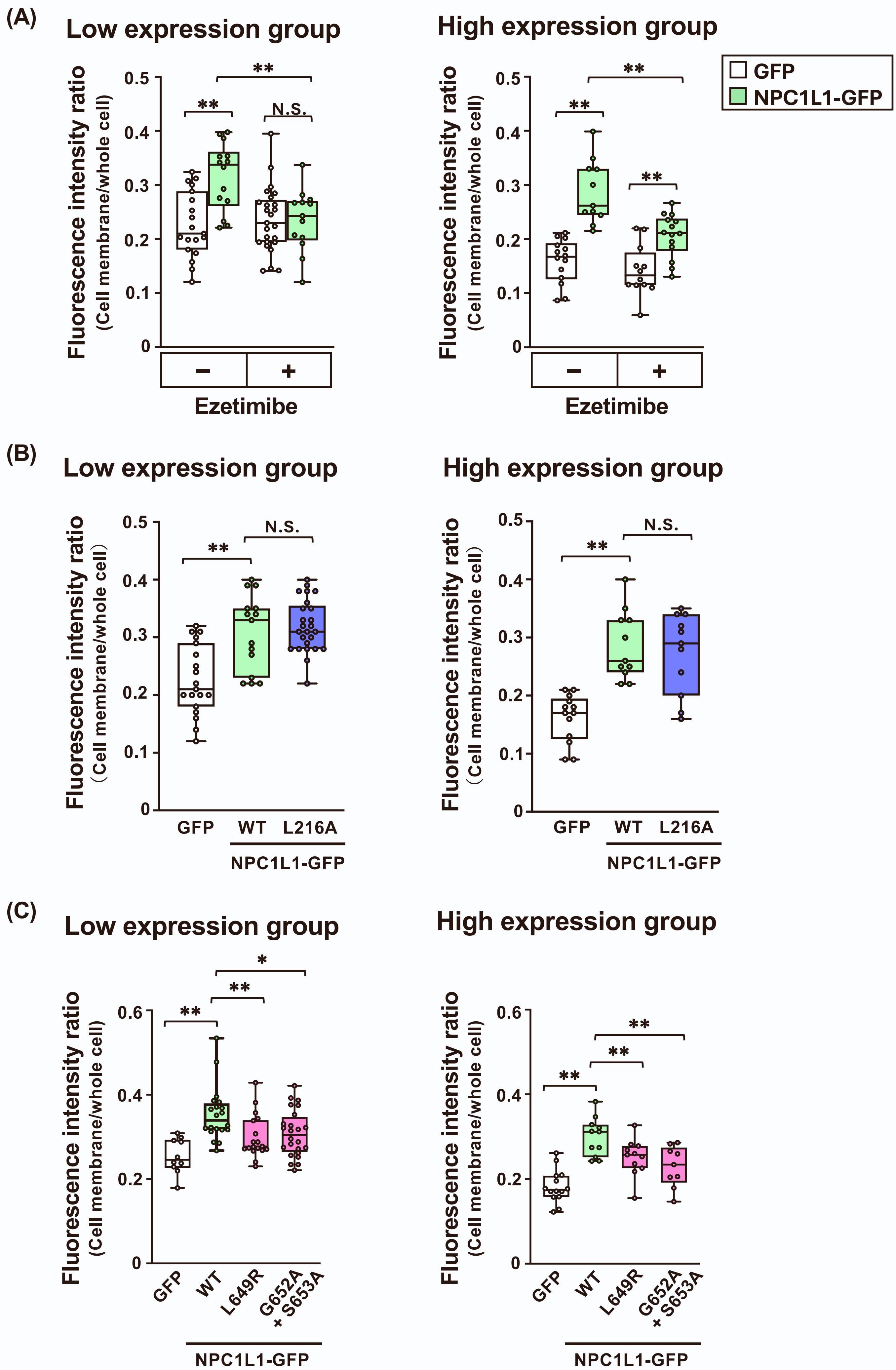

(A)
Low expression group
High expression group
GFP
NPC1L1-GFP
Fluorescence intensity ratio
(Cell membrane/whole cell)
0.5
0.4
0.3
0.2
0.1
0
**
**
N.S.
–
+
Ezetimibe
(B)
Low expression group
High expression group
N.S.
GFP
WT
L216A
NPC1L1-GFP
(C)
Low expression group
High expression group
0.6
*
L649R
G652A + S653A

(D)

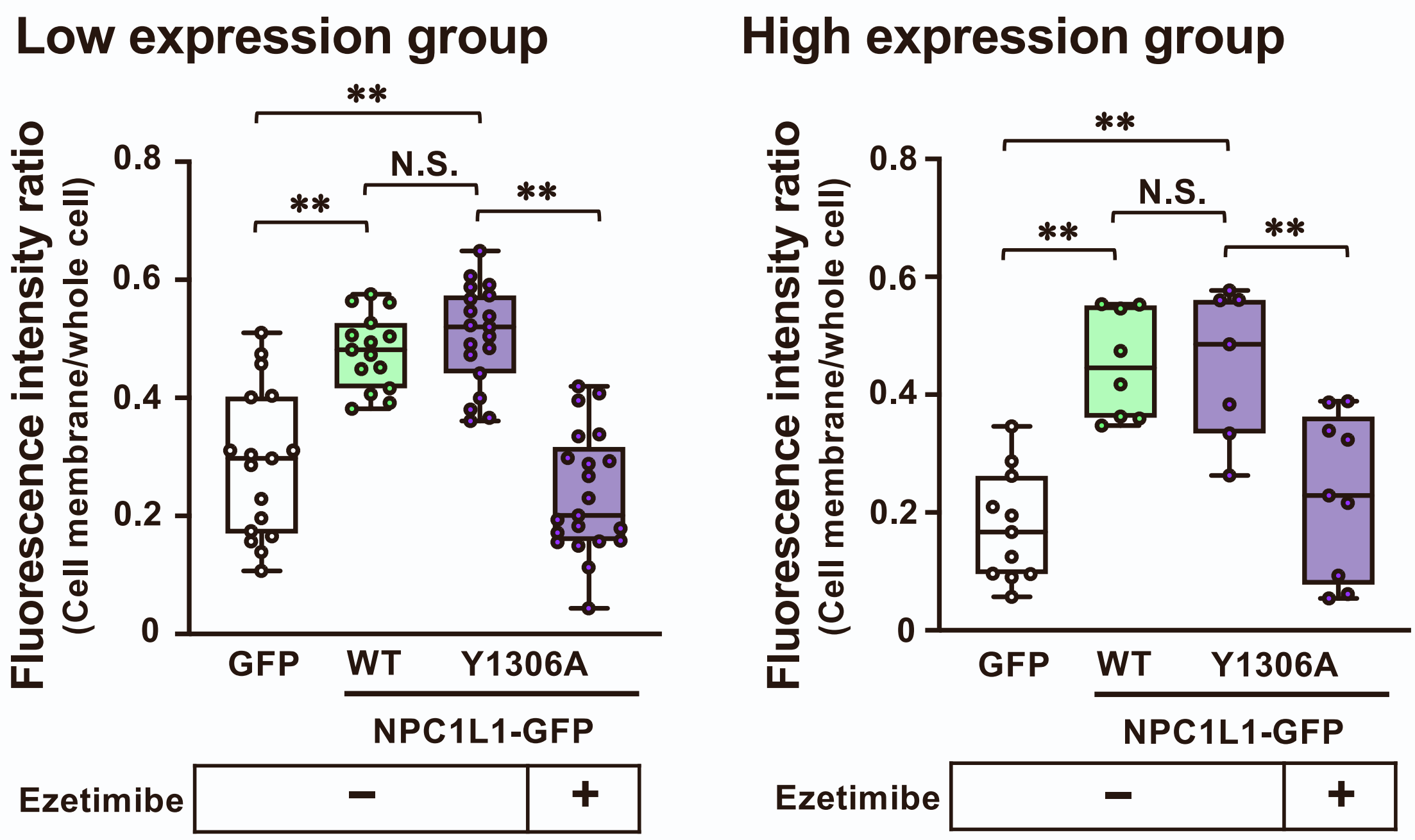

Figure S7. Fluorescence intensity ratio of mCherry-D4H classified by mCherry-D4H expression levels. The fluorescence intensity ratio of mCherry-D4H was analyzed separately according to whole-cell mCherry-D4H fluorescence levels. Cells with whole-cell fluorescence intensity lower than the mean value of all specimens in each assay were classified as the low expression group (left panels), whereas those with higher values were classified as the high expression group (right panels). Data from (A) Figure 1D, (B) Figure 3G, (C) Figure 5G, and (D) Figure 6G were analyzed. Box and whiskers plots represent median (line in box center), first and third quartile (lower and upper box border, respectively), and minimum and maximum values (whiskers). Dots represent individual data points. *$p<0.05$, **$p<0.01$, significantly different using Sidak's multiple comparison test. N.S., not significantly different.

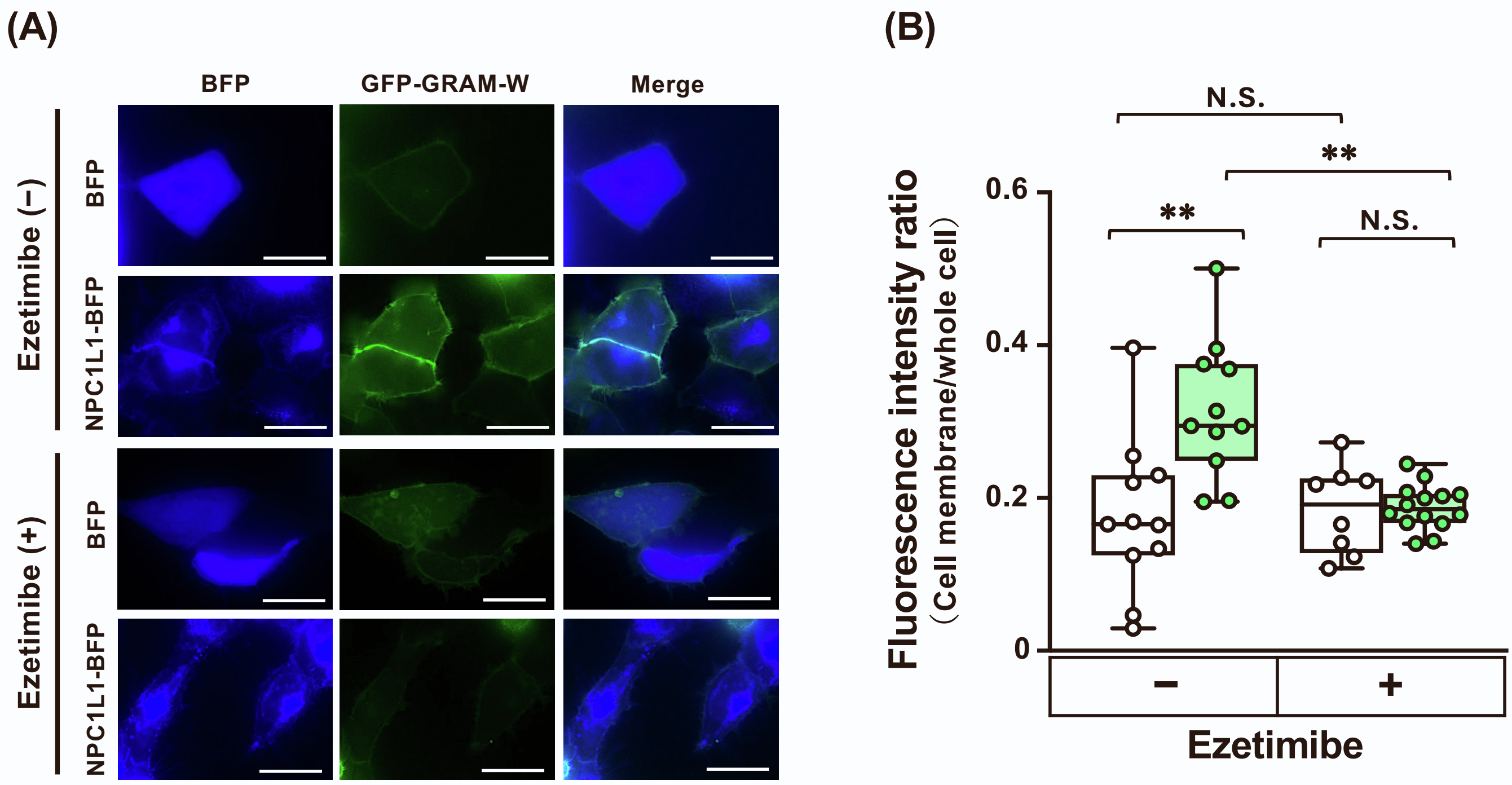

Figure S8. Effect of NPC1L1 on inner leaflet accessible cholesterol assessed using the GFP-GRAM-W probe. (A) McA-RH7777 cells were co-transfected with GFP-GRAM-W-expressing vector and either BFP- or NPC1L1-BFP-expressing vector. Twenty-four hours after transfection, the cells were further incubated for 24 h in the absence (−) or presence (+) of 30 μM ezetimibe. Representative images of BFP fluorescence, GFP fluorescence, and merged composites taken 48 h after transfection are shown. Scale bars represent 20 μm. (B) Quantification of GFP fluorescence intensity at the PM. Ratio of fluorescence intensity calculated as GFP fluorescence intensity at the PM divided by that of the whole cell is shown (n=8-14). Box and whiskers plots represent median (line in box center), first and third quartile (lower and upper box border, respectively), and minimum and maximum values (whiskers). Dots represent individual data points. **p<0.01, significantly different using Sidak's multiple comparison test. N.S., not significantly different.

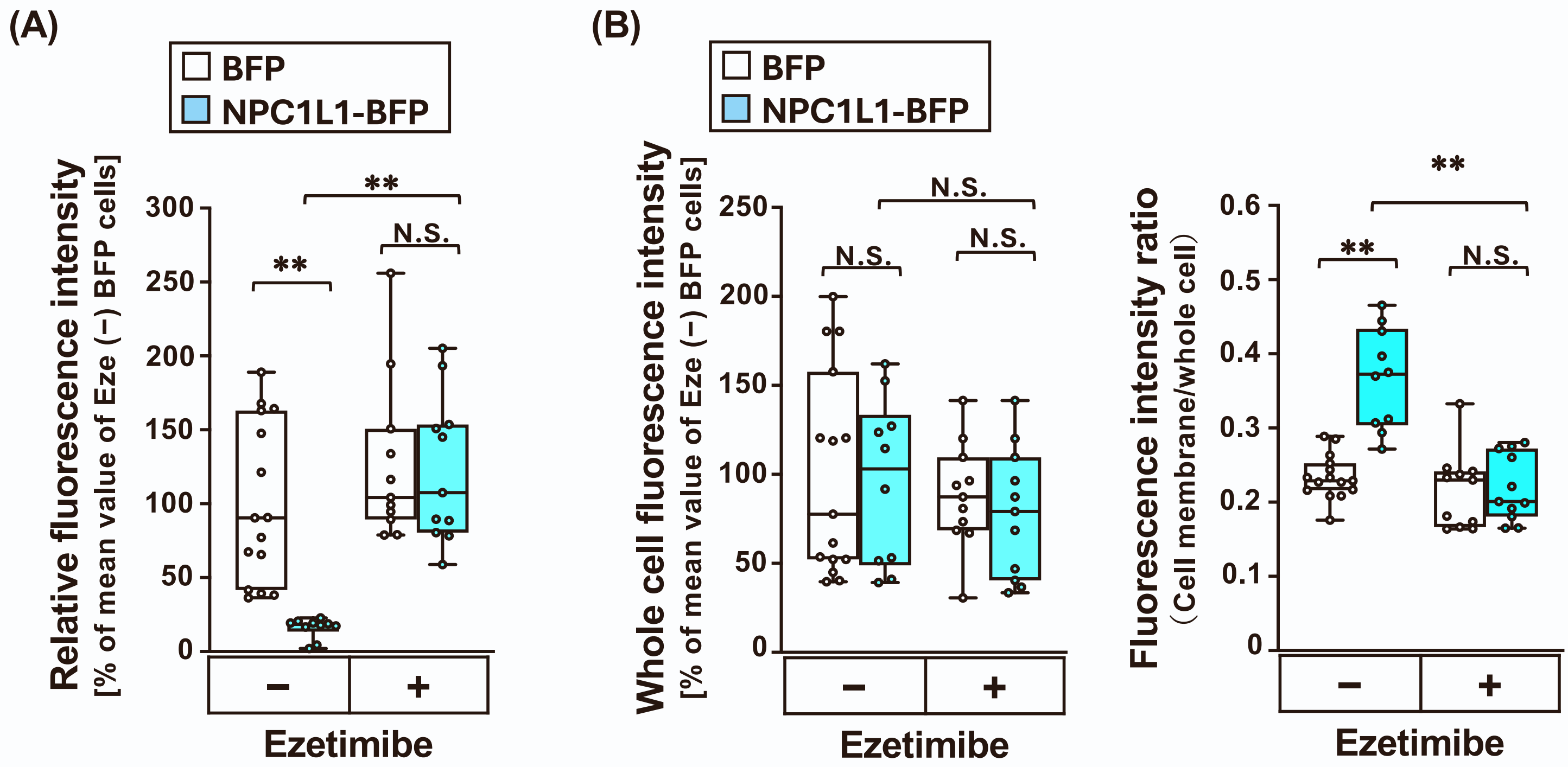

Figure S9. Quantitative comparison of accessible cholesterol distribution between lipid bilayers of the plasma membrane in McA-RH7777 cells under simultaneous labeling with GFP-D4 and mCherry-D4H probe. McA-RH7777 cells were co-transfected with mCherry-D4H-expressing vector and either BFP- or NPC1L1-BFP-expressing vector. Twenty-four hours after transfection, the cells were further incubated for 24 h in the absence (−) or presence (+) of 30 μM ezetimibe, followed by labeling with the recombinant GFP-D4 probe for 15 min. (A) Quantification of intensity of GFP fluorescence on the plasma membrane (PM). Relative fluorescence intensity compared with the mean value of ezetimibe-untreated BFP-expressing cells [Eze (–), BFP cells] is shown (n=10-15). (B) Quantification of intensity of mCherry fluorescence in the whole cell and at the PM (n=10-15). Relative whole cell fluorescence intensity compared with the mean value of ezetimibe-untreated BFP-expressing cells [Eze (–), BFP cells] is shown (left panel). Ratio of fluorescence intensity calculated as mCherry fluorescence intensity at the PM divided by that of the whole cell is shown (right panel). Box and whiskers plots represent median (line in box center), first and third quartile (lower and upper box border, respectively), and minimum and maximum values (whiskers). Dots represent individual data points. $^{**}p<0.01$, significantly different using Sidak's multiple comparison test. N.S., not significantly different.

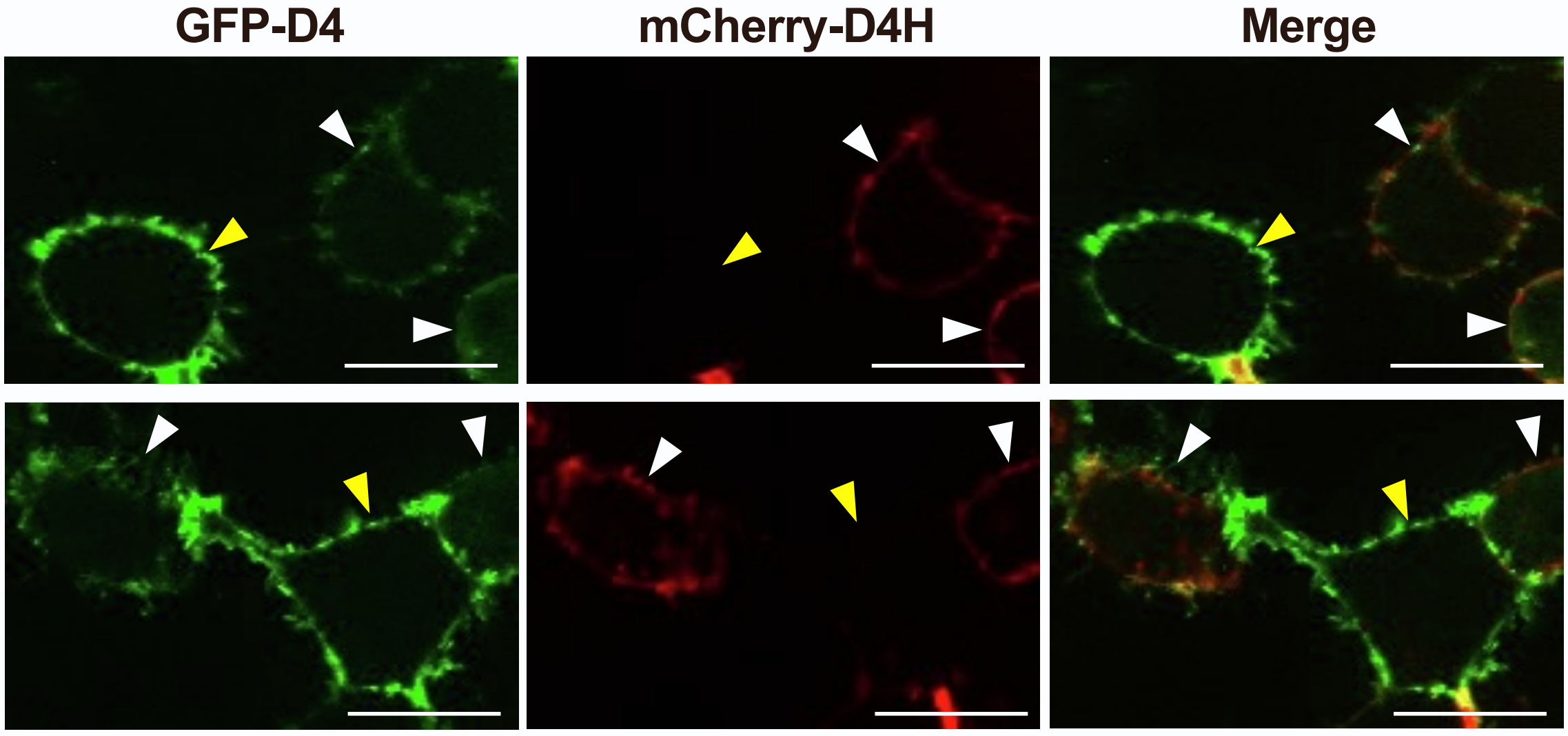

Figure S10. Simultaneous staining of accessible cholesterol in the outer and inner leaflets of the plasma membrane. McA-RH7777 cells were transfected with mCherry-D4H-expressing vector. Forty-eight hours after transfection, the cells were labeled with the recombinant GFP-D4 probe for 15 min. Representative images of GFP fluorescence, mCherry fluorescence, and merged composites from two different fields of view are shown. Scale bars represent 20 µm. White arrowheads indicate cells expressing mCherry-D4H, whereas yellow arrowheads indicate cells with little or no mCherry-D4H expression.

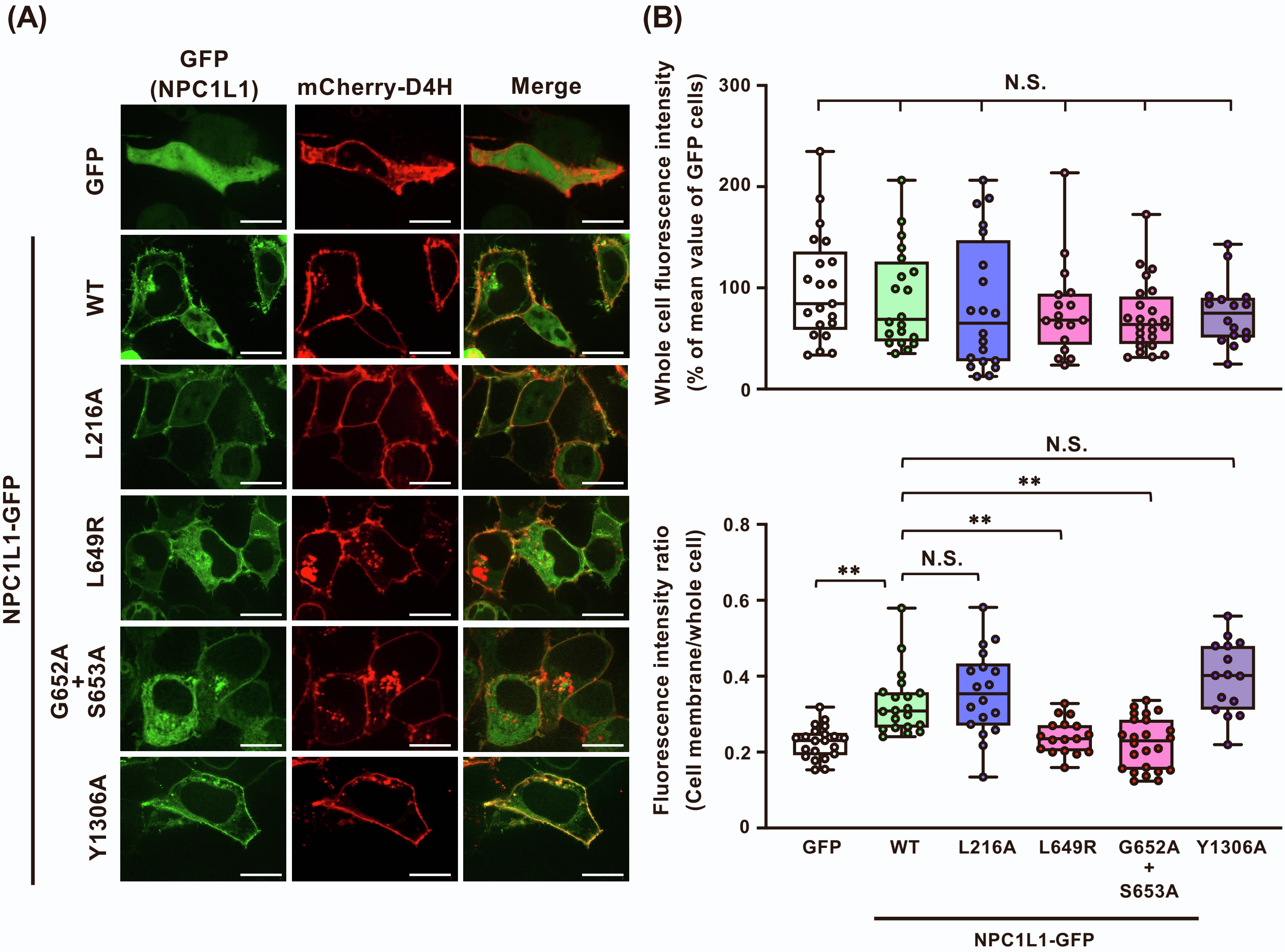

Figure S11. Live-cell imaging of inner leaflet cholesterol using mCherry-D4H probe. (A) McA-RH7777 cells were co-transfected with mCherry-D4H-expressing vector and either GFP-, NPC1L1^{WT}-GFP-, NPC1L1^{L216A}-GFP-, NPC1L1^{L649R}-GFP- or NPC1L1$^{G652A+S653A}$-GFP, or NPC1L1^{Y1306A}-GFP-expressing vector. Forty-eight hours after transfection, the cells were examined by fluorescence microscopy under live-cell conditions. Representative images of GFP fluorescence, mCherry fluorescence, and merged composites are shown. Scale bars represent 20 µm. (B) Quantification of mCherry fluorescence intensity in the whole cell and at the plasma membrane (PM) (n=15-24). Relative whole cell fluorescence intensity compared with the mean value of GFP-expressing cells is shown (top panel). Ratio of fluorescence intensity calculated as mCherry fluorescence intensity at the PM divided by that of the whole cell is shown (bottom panel). Box and whiskers plots represent median (line in box center), first and third quartile (lower and upper box border, respectively), and minimum and maximum values (whiskers). Dots represent individual data points. **$p<0.01$, significantly different using Sidak's multiple comparison test. N.S., not significantly different.

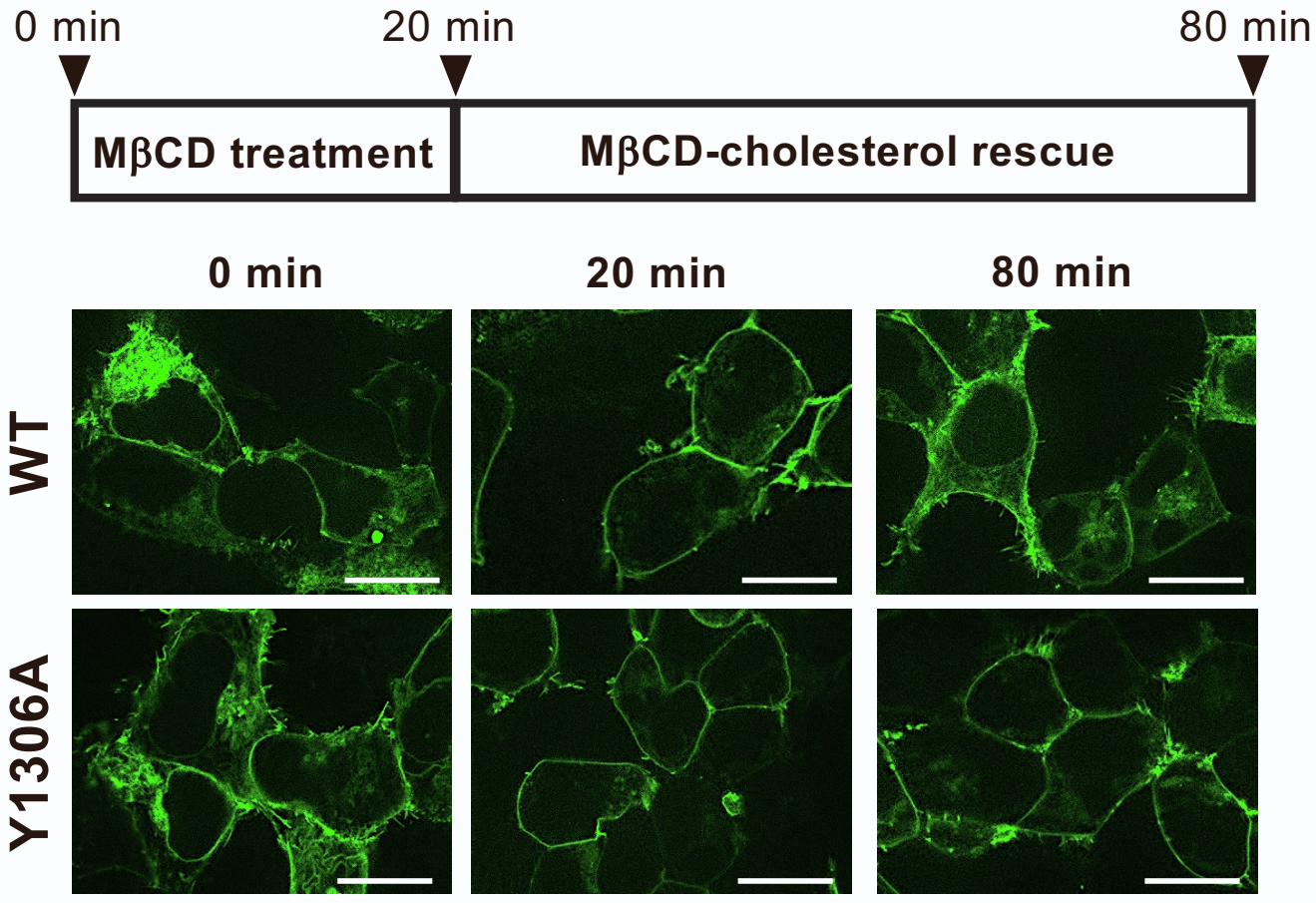

Figure S12. Cholesterol-dependent redistribution of wild-type and Y1306A mutant NPC1L1. McA-RH7777 cells transfected with either wild-type (WT) NPC1L1-GFP- or Y1306A mutant NPC1L1-GFP-expressing vector were incubated in serum-free medium containing 1.5% methyl-β-cyclodextrin (MβCD) for 20 min to deplete cellular cholesterol. Cells were subsequently incubated with MβCD-complexed cholesterol (100 μM) for up to 60 min to rescue cellular cholesterol levels. Representative fluorescence microscopy images showing the subcellular localization of NPC1L1-GFP at the indicated time points are shown. Scale bars represent 20 μm.

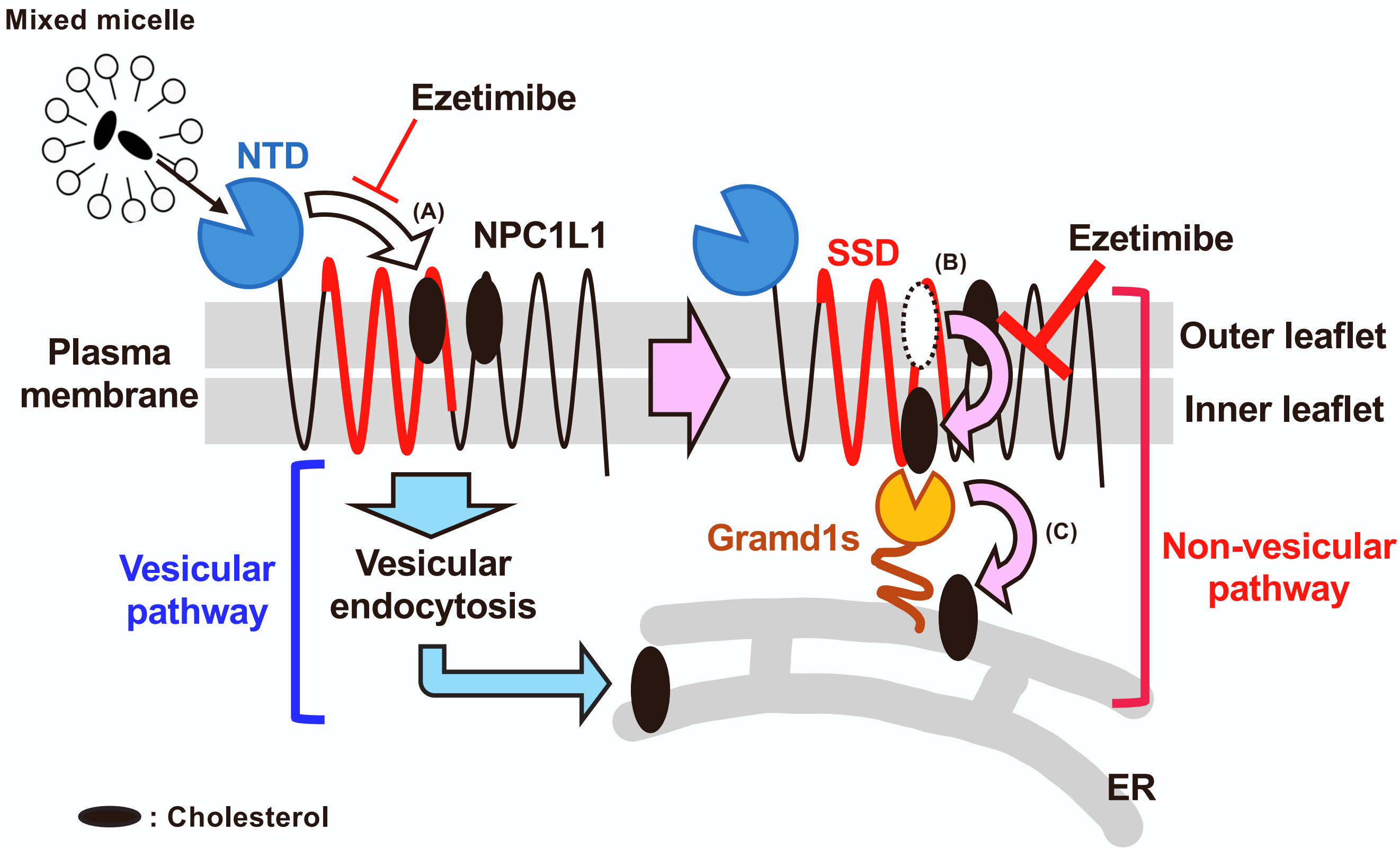

Figure S13. Proposed model of NPC1L1-mediated cholesterol transport from mixed micelles to the endoplasmic reticulum. Previous studies have established vesicular pathways for NPC1L1-mediated cholesterol uptake [S1-4]. The present study proposes an additional non-vesicular pathway that may operate independently of or in parallel with these established mechanisms. (A) The N-terminal domain (NTD) of NPC1L1 directly binds micellar cholesterol and facilitates its insertion into the outer leaflet of the plasma membrane. Ezetimibe partially inhibits this process. (B) The sterol-sensing domain (SSD) promotes redistribution of accessible cholesterol from the outer to inner leaflet of the plasma membrane, a process that is strongly inhibited by ezetimibe. (C) Accessible cholesterol in the inner leaflet is subsequently transferred to the endoplasmic reticulum (ER) by intracellular lipid transfer proteins such as Gramd1 family proteins (Gramd1s), as reported previously [S5].

References

S1. Ge, L., Qi, W., Wang, L.J., Miao, H.H., Qu, Y.X., Li, B.L., and Song, B.L. (2011). Flotillins play an essential role in Niemann-Pick C1-like 1-mediated cholesterol uptake. Proc Natl Acad Sci U S A *108*, 551-556. 10.1073/pnas.1014434108.

S2. Xie, C., Li, N., Chen, Z.J., Li, B.L., and Song, B.L. (2011). The small GTPase Cdc42 interacts with Niemann-Pick C1-like 1 (NPC1L1) and controls its movement from endocytic recycling compartment to plasma membrane in a cholesterol-dependent manner. J Biol Chem *286*, 35933-35942. 10.1074/jbc.M111.270199.

S3. Li, P.S., Fu, Z.Y., Zhang, Y.Y., Zhang, J.H., Xu, C.Q., Ma, Y.T., Li, B.L., and Song, B.L. (2014). The clathrin adaptor Numb regulates intestinal cholesterol absorption through dynamic interaction with NPC1L1. Nat Med *20*, 80-86. 10.1038/nm.3417.

S4. Xiao, J., Dong, L.W., Liu, S., Meng, F.H., Xie, C., Lu, X.Y., Zhang, W.J., Luo, J., and Song, B.L. (2023). Bile acids-mediated intracellular cholesterol transport promotes intestinal cholesterol absorption and NPC1L1 recycling. Nat Commun *14*, 6469. 10.1038/s41467-023-42179-5.

S5. Ferrari, A., Whang, E., Xiao, X., Kennelly, J.P., Romartinez-Alonso, B., Mack, J.J., Weston, T., Chen, K., Kim, Y., Tol, M.J., et al. (2023). Aster-dependent nonvesicular transport facilitates dietary cholesterol uptake. Science *382*, eadf0966. 10.1126/science.adf0966.

Supplemental data

Figure 3B

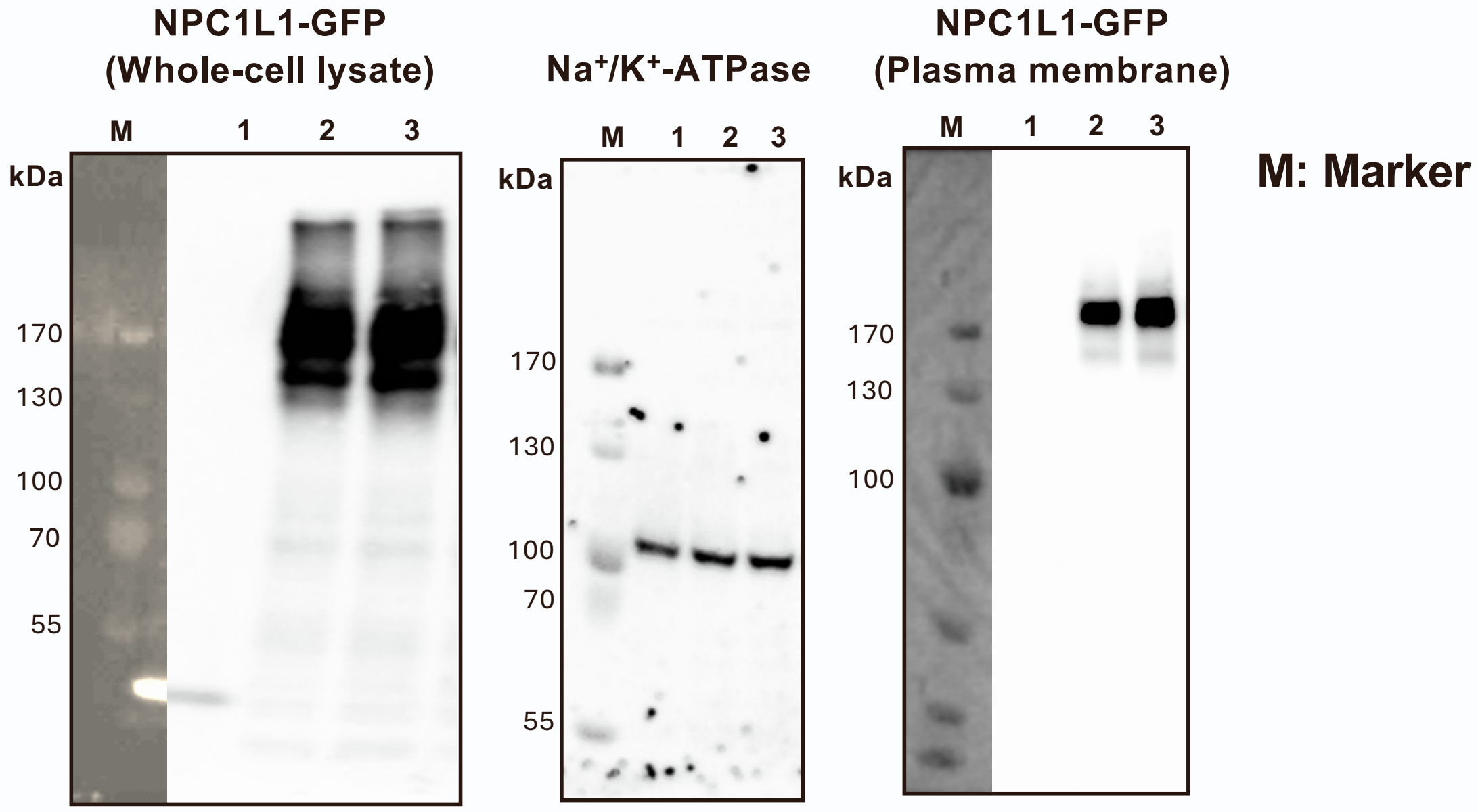

Figure 5B

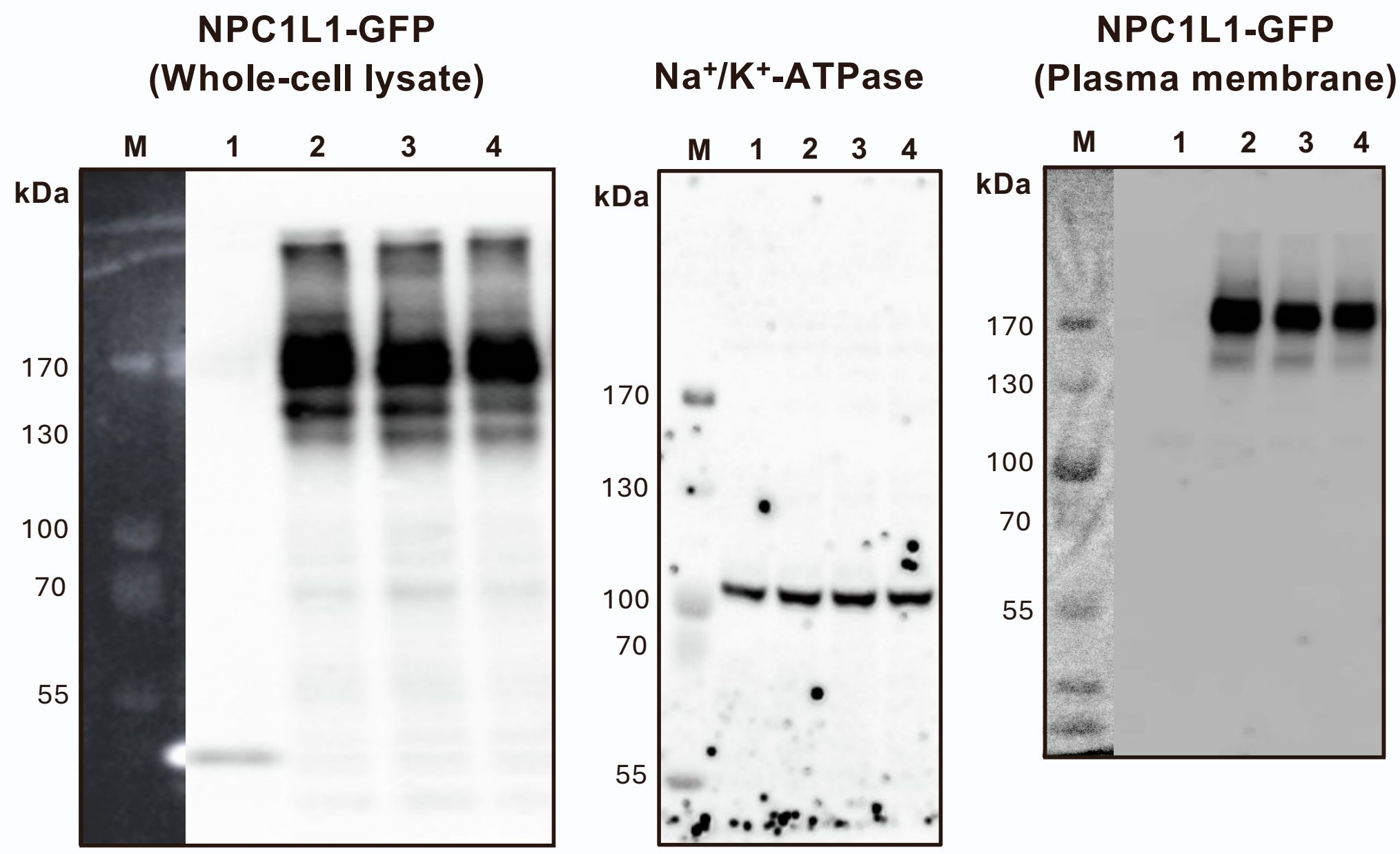

Figure 6B

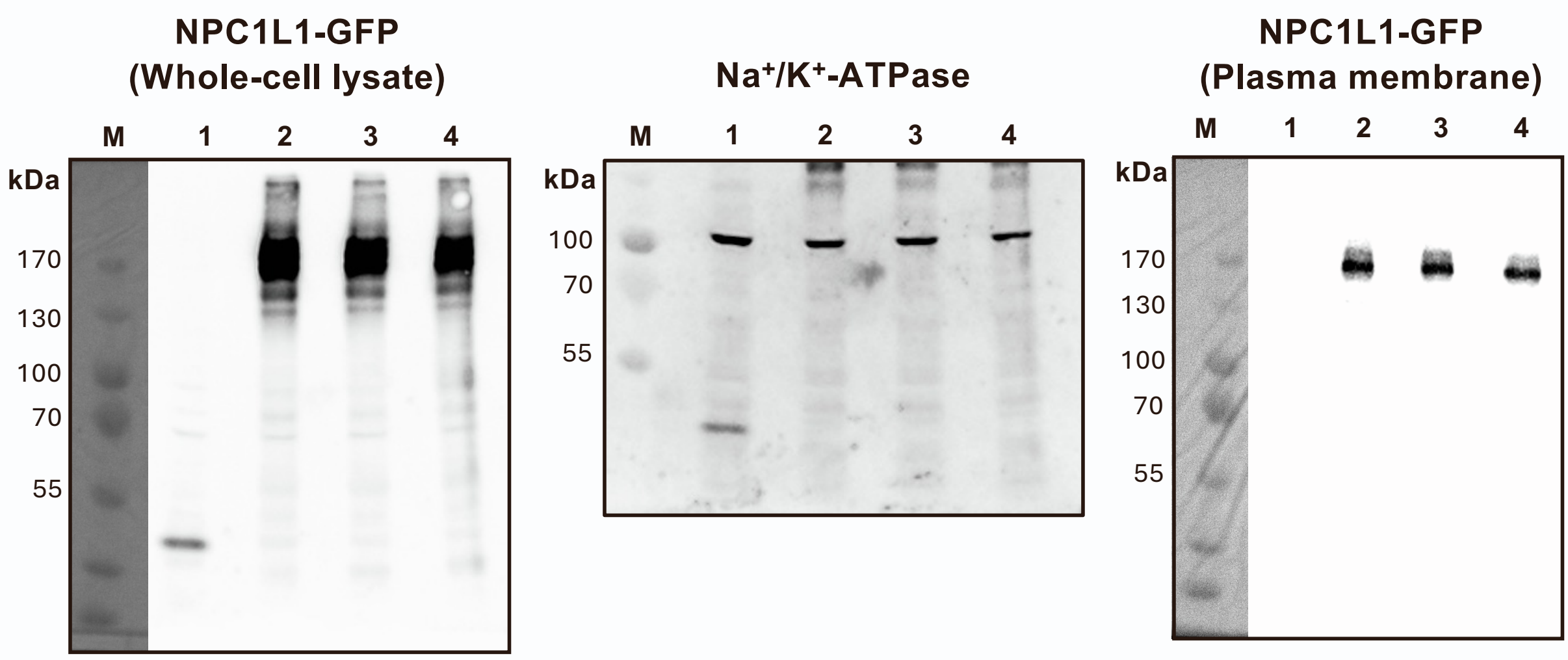

Figure 7B

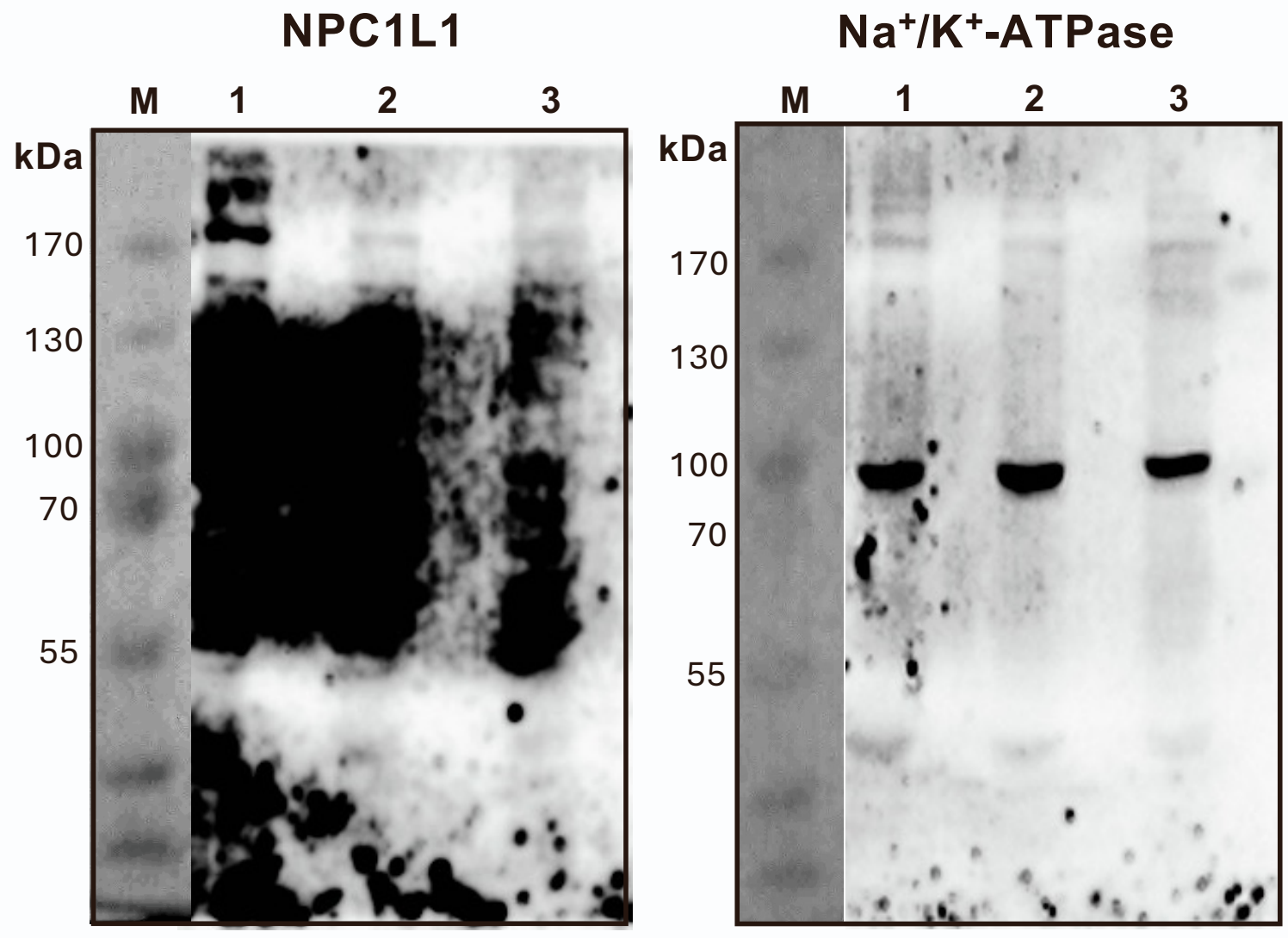

Figure S4

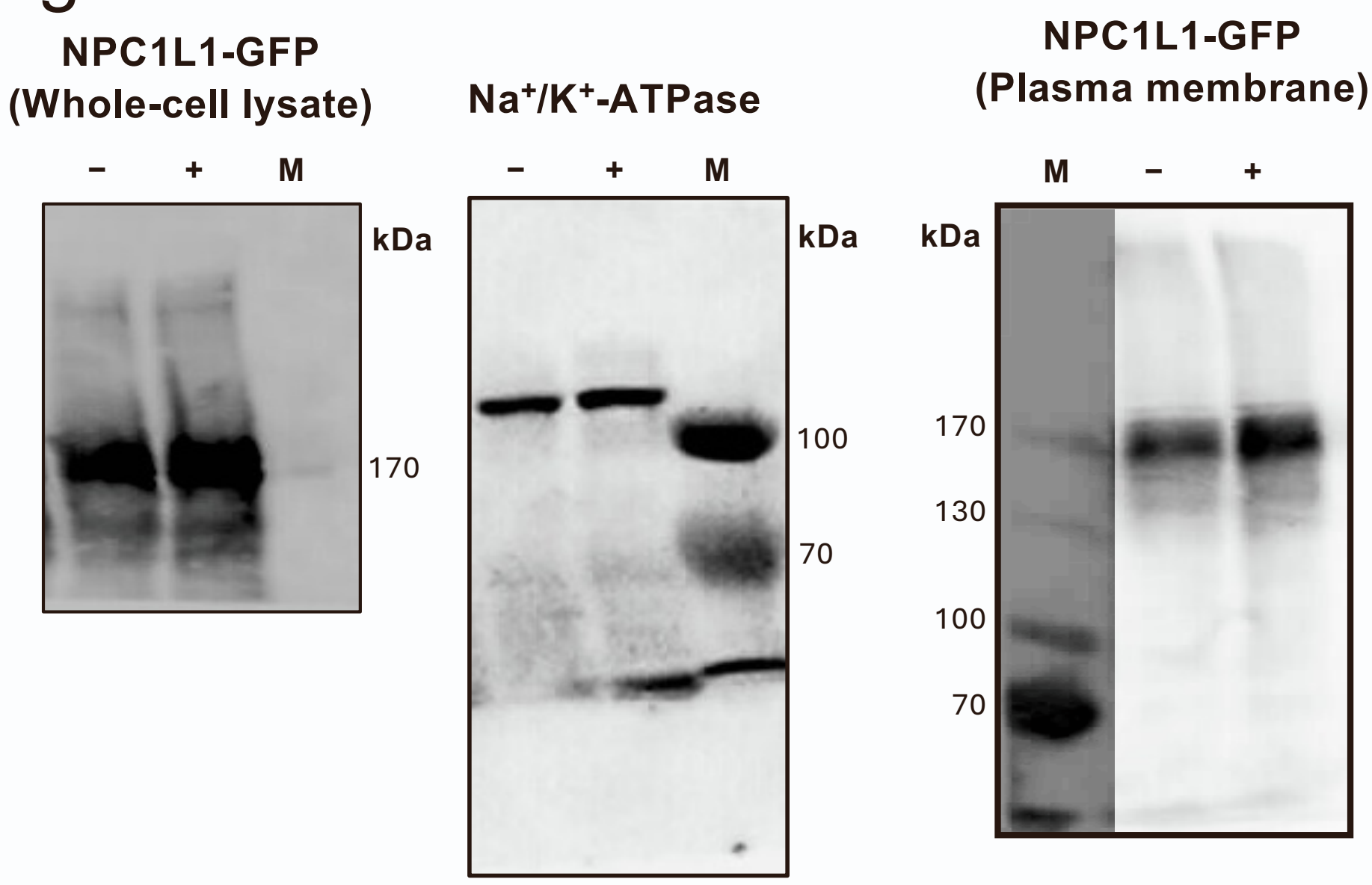

Data S1. Uncropped western blot images. Uncropped western blot images corresponding to main figures 3B, 5B, 6B, 7B, and supplemental figure 4 are shown.
